# Spanish-Language Consumer Health Information Technology Interventions: A Systematic Review

**DOI:** 10.2196/jmir.5794

**Published:** 2016-08-10

**Authors:** Alexis V Chaet, Bijan Morshedi, Kristen J Wells, Laura E Barnes, Rupa Valdez

**Affiliations:** ^1^ Department of Public Health Sciences University of Virginia Charlottesville, VA United States; ^2^ Department of Psychology San Diego State University San Diego, CA United States; ^3^ Department of Systems and Information Engineering University of Virginia Charlottesville, VA United States

**Keywords:** health information technology, consumer health information, consumer health informatics, health education, health promotion, health care quality, access, and evaluation, patient compliance, patient participation, patient satisfaction, patient preference, patient education, preventive health services, Hispanic, Latinos, cultural characteristics, cultural competency, ethnicity

## Abstract

**Background:**

As consumer health information technology (IT) becomes more thoroughly integrated into patient care, it is critical that these tools are appropriate for the diverse patient populations whom they are intended to serve. Cultural differences associated with ethnicity are one aspect of diversity that may play a role in user-technology interactions.

**Objective:**

Our aim was to evaluate the current scope of consumer health IT interventions targeted to the US Spanish-speaking Latino population and to characterize these interventions in terms of technological attributes, health domains, cultural tailoring, and evaluation metrics.

**Methods:**

A narrative synthesis was conducted of existing Spanish-language consumer health IT interventions indexed within health and computer science databases. Database searches were limited to English-language articles published between January 1990 and September 2015. Studies were included if they detailed an assessment of a patient-centered electronic technology intervention targeting health within the US Spanish-speaking Latino population. Included studies were required to have a majority Latino population sample. The following were extracted from articles: first author’s last name, publication year, population characteristics, journal domain, health domain, technology platform and functionality, available languages of intervention, US region, cultural tailoring, intervention delivery location, study design, and evaluation metrics.

**Results:**

We included 42 studies in the review. Most of the studies were published between 2009 and 2015 and had a majority percentage of female study participants. The mean age of participants ranged from 15 to 68. Interventions most commonly focused on urban population centers and within the western region of the United States. Of articles specifying a technology domain, computer was found to be most common; however, a fairly even distribution across all technologies was noted. Cancer, diabetes, and child, infant, or maternal health were the most common health domains targeted by consumer health IT interventions. More than half of the interventions were culturally tailored. The most frequently used evaluation metric was behavior/attitude change, followed by usability and knowledge retention.

**Conclusions:**

This study characterizes the existing body of research exploring consumer health IT interventions for the US Spanish-speaking Latino population. In doing so, it reveals three primary needs within the field. First, while the increase in studies targeting the Latino population in the last decade is a promising advancement, future research is needed that focuses on Latino subpopulations previously overlooked. Second, preliminary steps have been taken to culturally tailor consumer health IT interventions for the US Spanish-speaking Latino population; however, focus must expand beyond intervention content. Finally, the field should work to promote long-term evaluation of technology efficacy, moving beyond intermediary measures toward measures of health outcomes.

## Introduction

Patients are at the heart of the health care system. As primary stakeholders, they are not only affected by national and local policy, medical services, and the health care workforce, but also have the ability to affect health care cost, quality, and access through individual and community engagement. Patient engagement is a broadly defined term used to describe patient acquisition of knowledge, skills, ability, and motivation to participate in positive health behaviors and the interventions increasing these attributes [1,2]. A wide range of factors including literacy level, personal interest, information quality and access, and knowledge impact a patient’s ability to engage in their health and health care [3-7]. Engagement not only increases a patient’s overall satisfaction with the health care experience but also directly impacts health outcomes [8,9]. Engaged patients are more proactive in using preventative health resources such as screenings and immunizations, more willing to ask questions of their provider, more effective in managing chronic conditions, and less likely to participate in unhealthy lifestyle behaviors such as smoking and drug use [10-12]. As the complexity of medical technology and the burden of chronic disease grow, empowering patients to engage on all levels of care will be critical to reducing health care costs, improving population health, and maintaining patient satisfaction [13].

Consumer health information technology (IT) is increasingly being used to engage patients in shared decision making, self-management, and disease prevention through facilitation of health information access, social and clinical support, and electronic communication [14,15]. Consumer health IT may be conceptualized within the field of wellness informatics, which Grinter et al define as “a human-centered computing science focused on the design, deployment, and evaluation of human-facing technological solutions to promote and manage wellness acts, such as the prevention of disease and the management of health” (pg. 78) [16]. Although consumer health IT has varied definitions [15,17-19], there is a consensus that it is a form of electronic technology used by lay people to support health and health care management. Consumer health IT platforms include a variety of electronic systems such as desktop or laptop computers, touchscreen kiosks, personal wireless devices, and mass media [16,18,20,21]. A growing body of research has explored the feasibility, acceptability, and efficacy of these tools in promoting health [15,22-26]. While challenges remain in the standardized evaluation and cost-benefit analysis of consumer health IT [27,28], initial studies have shown potential for these engagement-oriented tools to positively impact health outcomes, quality of life, hospital readmission rates, and mortality [29-34].

Critical to the design of technologies that facilitate health and health care management is the consideration of population needs and characteristics [35-38]. A patient-centered design approach aims to systematically partner with patients in the creation and tailoring of technologies to best suit their unique needs, skills, environments, and preferences [36,39-41]. Design of technologies without consideration of the end user can lead to user frustration, error and misuse, and technology abandonment [41-44].

As consumer health IT becomes more thoroughly integrated into patient care, it is critical that these tools are appropriate for the diverse patient populations whom they are intended to serve [45-48]. Cultural differences associated with ethnicity are one aspect of diversity that may play a role in user-technology interactions [30,46,49]. Many studies that focus on consumer health IT within the literature do not have a diverse population sample [18]. It is difficult to determine whether lessons learned from heterogeneous populations may be universally applicable to minority ethnic groups. Instead there may be unique lessons to be learned. While there has been growing recognition of the importance of providing culturally competent health care [50], most of these initiatives have focused on providers, health care organizations, and public health interventions [51]. Given the potential for consumer health IT to enhance patient well-being both within and outside the institutional health care setting, it is critical that we view the design of these tools through the same conscientious lens of cultural competency [52].

Research within this field is both timely and important because of existing health disparities faced by ethnic minority populations and the national priority to decrease these disparities [53]. Designing appropriate consumer health IT interventions for ethnic minorities offers an opportunity to bridge existing health disparities faced by these populations [53]. In contrast, failure to design consumer health IT appropriately for ethnic minority groups risks exacerbating this divide [54]. Consequently, there is a need to assess our current understanding of the intersection between culture, ethnicity, and consumer health IT and to elucidate areas for future research within the field.

This paper focuses on a single ethnic group: the US Spanish-speaking Latino population, a heterogeneous group consisting of numerous subpopulations. This ethnic population was chosen for its current and increasing prominence within the United States. The Latino population represents the nation’s largest ethnic minority, numbering over 54 million [55]. In accordance with other ethnic minorities, Latinos face stark disparities in health and health literacy, influenced by poverty, institutional racism, and linguistic barriers, among other factors [56]. English-speaking Latinos show higher levels of technology ownership than their Spanish-speaking counterparts [57], and the latter group experiences worse self-reported health status and access to care [58]. This study aims to evaluate the current scope of consumer health IT targeted to the US Spanish-speaking Latino population, characterizing interventions in terms of technological attributes, health domains, cultural tailoring, and evaluation methods. In doing so, it identifies gaps in and limitations of interventions for this specific population and offers considerations for research within the broader field of culturally informed consumer health IT [46].

## Methods

### Design

A narrative synthesis [59] was conducted of existing Spanish language consumer health IT interventions indexed within health and computer science databases. A narrative review is a qualitative systematic review, which looks for themes or constructs present within a body of studies. In contrast to meta-analysis, the aim of a narrative review is to create a broad understanding of a particular phenomenon [59]. The aim of this study was to synthesize existing literature and to systematically assess gaps in consumer health IT interventions tailored to US Spanish-speaking Latinos.

### Search Strategy

Searches were first conducted in August 2014 within four health sciences (ie, PubMed, Web of Science, CINAHL, Cochrane Central Register of Controlled Trials [Cochrane]) and three computer sciences and engineering databases (Compendex, IEEE Xplore, and the Computers and Applied Sciences Complete [CASC]). A second search was run in September 2015 within these databases to capture additional articles published during the screening process. All databases were accessed via the University of Virginia libraries. A third search was run in June 2016 to expand the search to a 25-year time span from 1990-2015. Search terms were divided into three clusters referencing technology, ethnicity, and patient-centeredness (see Table 1). Terms were adapted for each unique database in consultation with a University of Virginia librarian. The search was limited to English language articles with human subjects. Database-specific Boolean search strings can be seen in the Multimedia Appendix 1.

**Table 1:** Search terms for PubMed (terms were adapted for each database).

Technology	Ethnicity	Patient-centeredness
cellular phone^a^	Hispanic Americans	consumer health information
mobile phone	Hispanic	health education^a^
mobile computing	Spanish Americans	health promotion^a^
mobile health	Latino(a)	health care quality, access, and evaluation^a^
text messaging	Spanish-speaking	patient compliance
internet^a^		patient participation
ehealth		patient satisfaction
blogging		patient preference
social media		patient education
facebook		preventive health services^a^
twitter		
telemedicine^a^		
audio player		
audiovisual aids^a^		
multimedia		
health records, personal		
computer systems^a^		
tablet computer		
computer/utilization^a^		
user-computer interface^a^		
computer user		
television^a^		
radio^a^		
soap opera		
reminder system		
educational technology^a^		
medical informatics		
health information technology		

^a^Medical Subject Headings (MeSH) term.

The combined electronic searches identified 2742 records. Records were divided as follows: PubMed (1798 citations), Web of Science (42 citations), CINAHL (717 citations), Cochrane (87 citations), Compendex (6 citations), CASC (136 citations), and IEEE Xplore (0 citations). After removal of duplicates, a combined total of 2626 unique records was compiled for preliminary abstract review.

### Inclusion Criteria

The search was limited to full-text, English language articles published between January 1990 and September 2015, with additional inclusion and exclusion criteria described in Textbox 1. If inclusion could not be determined by information provided within the abstract, the article was included for full-text review. Full-text inclusion criteria were identical to those used in abstract screening.

**Inclusion criteria for abstract screening and full-text review.**

Article characteristics:

- Article must be published between January 1990 and September 2015.
- Article must be in the English language.

Population characteristics:

- Participants must live within the United States, defined by the 48 contiguous states, Alaska, and Hawaii.
- If participants lived in both the United States and abroad, article must analyze US participants as cohesive population subset.

Intervention characteristics:

- Intervention must involve electronic technology (technology using electricity). This includes radio, television, mobile phone, computer, tablet, MP3 player, etc.
- Information delivered through the intervention must be available in Spanish.
- Intervention must target health and include topics pertaining to one or more of the following:
  - disease treatment
  - disease prevention
  - health education
  - personal safety
  - access to care
  - personal wellness
  - mental health
  - well-being
  - care of dependents
- Patient and/or the patient’s legal guardian must be the end user and direct benefactor of the device.
- Interventions targeting providers are excluded.
- Interventions consisting only of phone calls were excluded.

### Study Selection

Study selection consisted of two steps: abstract screening and full-text review. Abstracts were independently screened by authors AC and BM and compared at intervals of 50-100 articles until a Cohen’s kappa score of .95, indicating near-perfect agreement [60], was reached after three rounds. Discrepancies were discussed and eligibility criteria were refined between intervals when necessary. After final inclusion criteria were agreed upon, the remaining abstracts were divided between AC and BM and screened independently.

**Figure 1:**
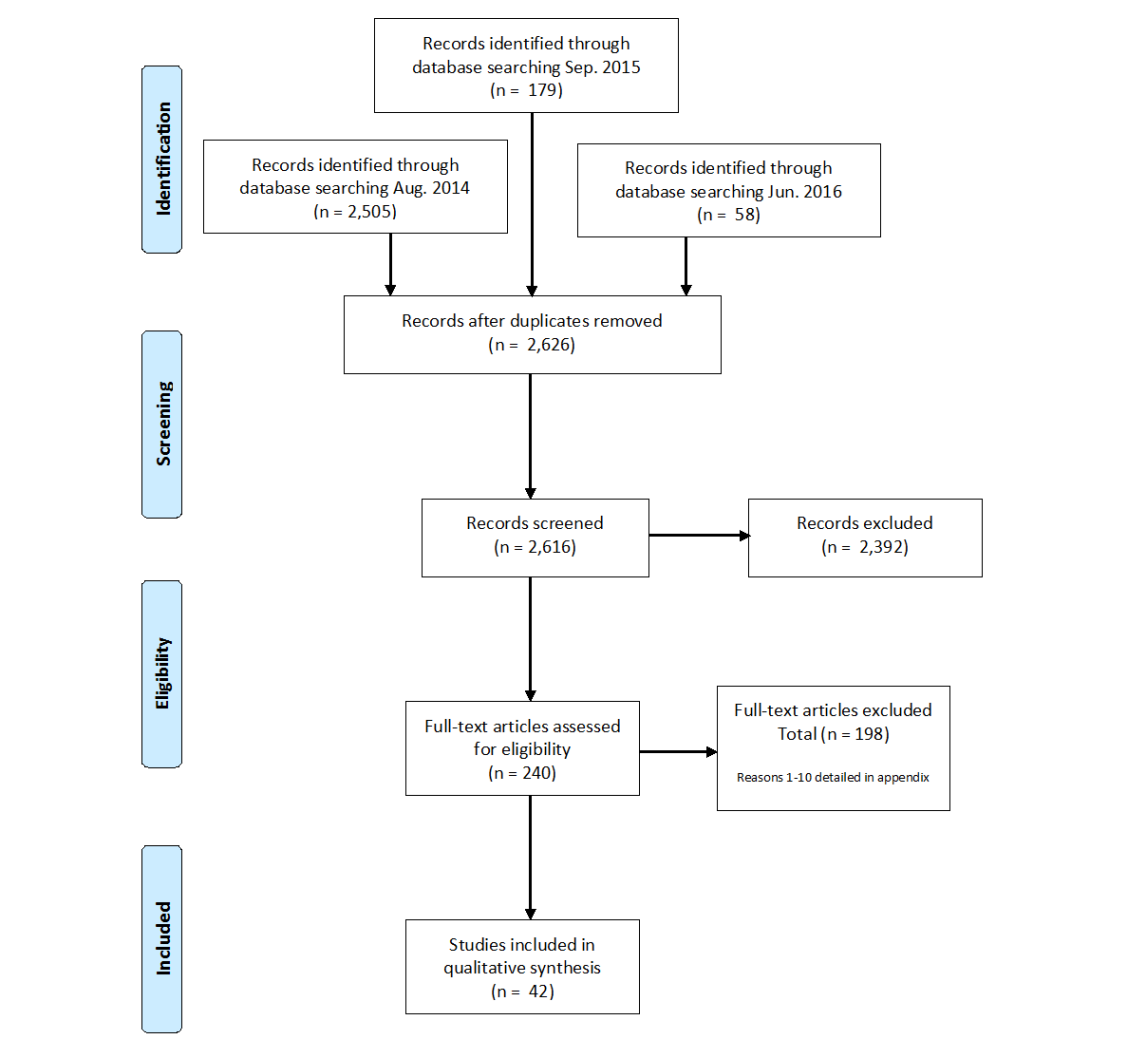
PRISMA schematic of study selection.

In total, 240 articles were returned for full-text review. AC reviewed all full-text articles using inclusion criteria. Reasons for article exclusion are detailed in the Multimedia Appendix 2, and excluded articles are listed in Multimedia Appendix 3. If an article failed multiple inclusion criteria, the first criterion identified upon a linear read of the article from introduction to conclusion was documented. The Preferred Reporting Items for Systematic Reviews and Meta-Analyses (PRISMA) [61] flowchart representing the study selection process is shown in Figure 1.

### Data Extraction

We identified 42 articles for full-text extraction. AC and BM independently reviewed and extracted data from all 42 articles (see Multimedia Appendix 4). A standardized template within Microsoft Excel was used to ensure systematic extraction of data. Extraction data were compared, and all discrepancies led to a joint article review and discussion. The following data were collected: (1) first author’s last name, (2) publication year, (3) population characteristics (eg, sample size, gender, age, percent Latino, ethnicity), (4) journal domain, (5) health domains, (6) technology platform, (7) technology functionality, (8) available languages of consumer health IT intervention, (9) US region, (10) cultural tailoring category, (11) population density (eg, urban versus rural), (12) intervention delivery location, (13) evaluation metrics, (14) evaluation results, and (15) study design.

#### Deductively Derived Categories

Categories for journal domain [62], US region [63], study design [64], cultural tailoring [46], and technology functionality [65] were deductively derived using external frameworks.The external frameworks for technology functionality and cultural tailoring categories are summarized in Table 2. Evaluation metrics were categorized according to a deductive framework developed by the collective expertise of the study team (see Table 4).

**Table 2:** Technology functionality framework [61].

Functionality subcategory	Definition
Inform	Provide information in a variety of formats (text, photo, video)
Instruct	Provide instructions to the user
Record	Capture user-entered data
Display	Graphically display user-entered data/ output user-entered data
Guide	Provide guidance based on user-entered information (eg, recommend a physician consultation or course of treatment)
Remind/Alert	Provide reminders to the user
Communicate	Provide communication with health care provider/ patients and/or provide links to social networks

**Table 3:** Culturally-informed design framework [46].

Cultural tailoring category	Description	Examples of cultural considerations
Content	Message being delivered through the technology	Origins and consequences of health conditions
		Norms related to diet, religion, and division of labor
		Alternative medicine
Functionality	Array of actions performed by the technology	Culturally specific health management behaviors
		Perception of privacy and health care decision making
		Preferences for information delivery and communication (eg, voice communication)
Technology platform	Technology hardware used to deliver the health intervention	Access and exposure to technology
		Use of hardware within target population
		Role of Internet
User interface	Presentation and organization of the content and functionality	Cultural symbols
		Language and dialect
		Spatial orientation
		Colors

**Table 4:** Evaluation metrics categories.

Category	Description
Behavior/ attitude change	Changes in the lifestyle, disease management, or attitude toward a health topic or behavior. These include measurements such as changes in disease screening rates, treatment compliance, medical care utilization, performance of self-care tasks, attitude toward organ donation, attitude toward breast cancer screening, and attitude toward alcohol use.
Knowledge retention	Any measurement of information taught through technology intervention. These include measurements such as knowledge of diabetes care, knowledge of disease prevention techniques, or knowledge of vaccination schedules.
Self-reported health marker	Self-reported health measures including depression scale rankings, pain rankings, self-efficacy, psychosocial functioning, or quality of life.
Biometric health marker	Quantitative measures of body function including HbA1c levels, blood pressure, glycemic control, and body mass index. Both clinic-generated and self-reported biometric health markers were included within this category.
Usability	Specific feedback regarding physical characteristics of technology, user interface, acceptability of technology, and perceived utility. These include measures such as ease of use, readability, ability of patient to relate to video characters, acceptability of video length, emotional appeal, and satisfaction with device.

#### Inductively Derived Categories

Using the data abstracted from the individual studies, the research team inductively derived categories for the following variables: technology platform, population density, health domains, evaluation results, and intervention delivery location (see Table 5). Additionally, we followed the procedure below:

Technology platform: When it was not possible to infer the specific technology platform, the article was coded as “unspecified.” This was most common for video interventions that did not specify whether the video was delivered through a computer, digital video disc (DVD) player, videocassette recorder (VCR), or tablet.

Population density: If it was possible to infer an urban or rural location from article language or study location, the article was coded accordingly. For example, an intervention conducted within a “city center” was coded as “urban.”

Health domain: Given the numerous health domains investigated by studies, we engaged in a second round of coding to reduce the number of health domain categories. For example, “infant immunization” was coded as “infant, child health, or maternal health.”

Intervention delivery location: Similarly, some intervention delivery locations were coded at a higher level of abstraction. For example, “mass media” and “internet” interventions were ultimately coded as “ubiquitous environment.”

Evaluation results: Results were drawn directly from the text of each article. If multiple outcomes were reported, only primary outcomes were included.

**Table 5:** Inductively derived categories.

Variable	Description
Population density	Article was categorized as “rural” only if authors specified a rural community. If authors did not use the term “urban” but specified a city or county that was predominantly urban, the article was classified as urban. If technology use occurred within an urban hospital center, the article was classified as urban. Articles that were unclear or did not specify any location were classified as “Did Not Specify (DNS).”
Intervention delivery locations	Clinic: Intervention delivery within a clinic, hospital, or medical center. Includes clinic waiting room or medical encounter.
	Ubiquitous environment: Intervention delivery could occur in multiple physical environments. Technologies accessed by patients through personal devices such as mobile phones, desktop computers, or radio, or through public mass media. This includes all interventions accessed through the Internet.
	Community center: Intervention delivery in any public gathering space that does not formally provide medical care (ie, not a clinic). This includes churches, schools, pharmacies, cafes, libraries, and other community centers.

### Data Analysis

Data were coded manually into numerical categories, and basic statistics were computed using Microsoft Excel Version 14.1. All percentages were calculated out of the total number of included studies (N=42). If a study did not report on a given category, the study was coded as “did not specify.” Studies could be categorized in multiple subcategories for the following: cultural tailoring category, evaluation metrics, technology platform and functionality, population density, intervention delivery location, and journal domain.

## Results

### General Study Characteristics

All studies were published between 1990 and 2015, with the majority of studies (30/42, 71%) published between 2009 and 2015. All 42 articles detailed distinct consumer health IT interventions [66-100, 122-124, 136-139]. Studies were published across 26 unique journal domains. Medical Sciences—general (15/42, 36%) and Medical Sciences—specialty (17/42, 40%) were the most prevalent subcategories, followed by Public Health and Safety (11/42, 24%). The mean sample size was 416 participants (SD 603; 42/42 articles). This value was inflated by the inclusion of four mass media studies that contained more than 1000 participants [92-95]. Randomized controlled trial (RCT; 18/42, 43%) and non-experimental (19/42, 45%) were the most commonly used study designs. The percentage of female study participants in the abstracted studies ranged from 49.5-100% (40/42), and the mean age of participants reported in the included studies ranged from 15.0-72.1 years (21/42). Across study samples, the percentage of Latino participants ranged from 51-100% (40/42). However, to be included in the study, it was required that studies have a majority of Latino participants. Country of origin was reported within half of the articles (21/42, 50%). Given inconsistent reporting strategies, however, it is difficult to make representative generalizations. Most studies focused within the western region of the United States (17/42, 41%) and on an urban population center (35/42, 83%).

### Intervention Characteristics

A wide variety of consumer health IT interventions have been used to target health within the Latino population. These interventions have focused most commonly on chronic diseases and included some degree of cultural tailoring. Computer, radio, and television were the most commonly used technology platforms; however, a fairly even distribution across all technologies was noted. Nearly all interventions had the functionality of informing the end user (38/42, 91%), and nearly one half of studies employed more than one functionality (19/42, 45%). The large majority of technology interventions (32/42, 76%) specified availability in English in addition to Spanish. Cancer (10/42, 24%), diabetes (9/42, 21%), and child, infant, or maternal health (9/42, 21%) were the most commonly addressed health domains. As shown in Table 6, a number of health domains were targeted by only one study; these included topics such as sexual health, appointment reminders, anesthesia, and health care utilization. The most common intervention delivery location was within a ubiquitous environment (19/42, 45%) or clinic setting (17/42, 41%). More than half of the interventions (25/42, 60%) were culturally tailored.

**Table 6:** Frequencies of selected intervention characteristics of included studies (for all domains, articles may be included within multiple subcategories).

		Frequency, n	Percentage, %
**Technology platform**			
	Computer	8	19
	Radio	8	19
	Television	8	19
	Kiosk	7	17
	Unspecified	5	12
	Mobile phone ‒ text message	5	12
	VCR	3	7
	DVD	2	4
	Tablet	2	4
	Not reported	0	0
**Intervention delivery location**			
	Ubiquitous environment	19	45
	Clinic setting	17	40
	Community center	5	12
	Not reported	2	5
**Population density**			
	Urban	35	83
	Rural	5	12
	Not reported	4	10
**Cultural tailoring characteristics**			
	Content	21	50
	User interphase	6	14
	Functionality	2	5
	Technology platform	1	2
	Not reported	17	40
**Technology functionality**			
	Inform	38	91
	Communicate	6	14
	Guide	5	12
	Instruct	4	10
	Record	4	10
	Remind/Alert	4	10
	Display	3	7
	Not reported	0	0
**Health domain**			
	Cancer	10	24
	Child, infant, or maternal health	9	21
	Diabetes	9	21
	Cardiovascular disease	3	7
	Organ donation	3	7
	Physical activity	2	5
	General adult health	2	5
	Sexual health	1	2
	Anesthesia	1	2
	Appointment reminder	1	2
	Driving under influence recidivism	1	2
	Pain	1	2
	Health care utilization	1	2
	Patient safety	1	2
	Not reported	0	0

### Evaluation and Results

Evaluation metrics included both intermediate health-related measures and measures of technology usability. Most articles (28/42, 67%) used more than one evaluation metric. The most commonly used evaluation metric subcategory was behavior/attitude change (31/42, 74%), followed by usability (20/42, 48%), and knowledge retention (22/42, 52%). Behavior/attitude change included metrics such as medication adherence [96,97], intention to become an organ donor [93], and colorectal cancer screening rate [98]. Knowledge retention included metrics such as knowledge of cardiovascular disease risk factors and prevention [99], knowledge of basic child immunization schedule [100], and knowledge of general recommendations for breast cancer treatment [95]. Specific details for each study, including evaluation results, can be found in Multimedia Appendix 7.

## Discussion

### Principal Findings

In summary, the Spanish language consumer health IT interventions targeting US Spanish-speaking Latinos published within the past 25 years (1990-2015) are characterized by a great amount of diversity with regards to technology platform and study design (eg, sample size and evaluation metrics); however, similarities can be seen in the technology functionality, specific populations, and health domains addressed by these interventions. Interventions most commonly focused on urban population centers, the western United States, and chronic health domains including cancer and diabetes. While sample size varied tremendously across studies, study samples were largely female. Behavior/attitude change, knowledge retention, and usability were the most commonly used evaluation metrics. Just over half of studies detailed some type of cultural tailoring, with content tailoring being the most common.

### Intervention Characteristics

#### Technology Platform and Functionality

A wide distribution of technology platforms to engage Spanish-speaking patients in their health and health care is seen within the literature. Computer, radio, and television were the most common platforms used across interventions. As we continue to explore newer technologies, it is important to understand what aspects of these intervention designs are applicable to other technology modalities. Moreover, as the technology platforms used to engage consumers evolve, researchers should be cognizant of disparities in technology exposure and access across subpopulations. For example, within the Latino population computer ownership as well as Internet usage, mobile phone ownership, smartphone ownership, and social media use vary significantly across age, socioeconomic status, and language dominancy [52]. For some subpopulations, traditional technologies may be more appropriate given varying levels of access and exposure to newer technology platforms.

Nearly all interventions served to inform or educate the end users while fewer interventions incorporated other functionalities such as delivering a direct service or treatment to patients. This is not surprising given that health education aims to equip patients with the knowledge and skills they need to manage their disease while promoting behavior change [101,102]. Nevertheless, future interventions could strive to incorporate additional functionalities to address specific user needs. For example, consumer health IT (whether publicly or privately disseminated) could be used to mitigate communication barriers between digitally underserved Latinos and health care providers or social resources [103].

#### Cultural Tailoring

This study reveals an initial movement toward integrating culturally tailored features into consumer health IT for the US Spanish-speaking Latino population. More than half of the articles mentioned some form of cultural tailoring, suggesting awareness of the interaction between culture and user interactions with technology. Intervention content was the predominant mechanism of tailoring, with fewer articles tailoring functionality, technology platform, or user interface. While there is abundant literature on culturally competent health care for Latinos in the past two decades [104-110], the cultural competency movement within the health sciences has focused predominantly on language, cultural traditions, and cultural differences in health beliefs within this population [111]; these domains are predominantly pertinent to technology content. In contrast, there have been some initial efforts within the fields of engineering, marketing, and health to shed light on the influence of cultural preferences not only on content, but also user interface, functionality, and technology platform [47,112-116]. Some of these studies have focused specifically on the Latino population [114-116]. Although these studies face some limitations in sample size, reliance on a conceptualization of cultural context as one broadly defined cultural identity, and lack a systematic understanding of the design space, they nonetheless provide initial design constructs to aid future consumer health IT designers in generating a more holistic understanding of culturally competent technology form and function preferences.

### Health Domains and Population Target

#### Health Domains

This study reveals a focus within the literature on chronic diseases and a need for future consumer health IT interventions to target two areas: underrepresented health domains within Latino subpopulations and challenges faced by Latinos in health care access and utilization. Given the disproportionate prevalence of diabetes, obesity, and cancer within the Latino population [112,117], focus on chronic disease prevention and management offers significant opportunity for social and economic incentives. Nonetheless, population health statistics can mask wider variation in Latino subpopulations. As a relevant example, Latino migrant farmworkers face higher concerns related to pesticide exposure given their living or work environment [118,119]. No articles included within this review targeted pesticide exposure, yet technology access within this population suggests health IT interventions may be feasible to address this topic. For example, mobile phone penetration among farmworkers has been found to be comparable to the general Latino population [120]. Furthermore, in cases where there is a lack of technology access, there are examples in the literature where technology was incorporated at the community level [28]. To avoid widening intragroup health disparities, future research should ensure that the needs of these subpopulations are met. In addition, beyond disease-specific health domains, future consumer health IT interventions for the US Spanish-speaking Latino population should target health care utilization. Lack of trust, economic barriers, lack of knowledge of services, immigration status, and linguistic and cultural differences contribute to a sense of disconnect between Latinos and the US health care system [121]. To mitigate these systemic barriers, future research should explore how consumer health IT interventions might be used to facilitate peer-support systems, connect patients to financial and linguistic services, or assist patients in health care system navigation.

#### Gender

It is important to note that the majority of participants across studies were female. This may be influenced by the fact that a large number of interventions focused within the category of “Child, Infant, or Maternal Health,” targeting health concerns such as breast cancer [122,123] or cervical cancer [124]. However, it is not uncommon for women to be more represented in scientific research [125,126].

#### Location

This study offers two principle findings regarding the location of intervention delivery. First, the majority of studies were conducted in urban settings, likely reflecting the location of academic institutions. Given that nearly 12% of the Latino population lives within a rural area [127] and that rural Latinos are less likely to receive preventative care, to be screened for certain cancers, and to meet vigorous physical activity recommendations than their urban counterparts [128], future intervention should engage rural communities to avoid exacerbating existing intragroup health disparities. Second, within urban populations, the vast majority of interventions were delivered through technology platforms that could be used in ubiquitous environments as opposed to commercial locations such as clinics or pharmacies. This likely reflects the increasing popularity of Internet-capable personal devices. Accelerating consumer health IT interventions built upon these platforms might allow for greater reach given the growing adoption of mobile phones and smartphone technologies within the Latino population [57]. Researchers, however, should be cognizant of wireless access limitations or unique structures of service provision within rural communities.

### Evaluation Metrics and Study Design

The majority of included studies focused on intermediate measures, or those that are conceptualized as precursors to predicting health outcomes, namely, knowledge retention and behavior/attitude change. Although studies have shown that positive behaviors can affect significant changes in chronic disease outcomes, these behaviors must be sustained in the long term for significant changes in health status [129]. Given documentation of high rates of attrition in the use of consumer health IT [130], future studies should address the longevity of these behavior/attitude changes. In addition, future studies should use both validated measures particular to a given health condition as well as validated measures that have relevance across health conditions such as the Patient Activation Measure (PAM) [12]. PAM assesses a patient’s knowledge, skill, and confidence for self-management of disease, which predicts health behaviors, self-management behaviors, and consumeristic type behaviors [12]. This will facilitate comparison of outcomes across consumer health IT interventions targeted to diverse health conditions within the US Spanish-speaking Latino population.

Given the interdisciplinary nature of studies focused on consumer health IT interventions for the US Spanish-speaking Latino population, diversity was seen both in types of evaluation metrics and combinations of metrics used by studies. Although this approach allows for multiple perspectives on effective intervention development, a key limitation is the ability to conduct meta-analyses and to compare findings across studies. Future research should synthesize various perspectives from relevant disciplines to create a framework for evaluation of consumer health IT for the US Spanish-speaking Latino population. A crosswalk approach might then be used to identify connections between various evaluation metrics [131]. The large number of studies that used an RCT design is a promising finding given that RCTs are considered to be the strongest form of clinical evidence within intervention-based studies [132]. The use of RCT study design is not uncommon within in the evaluation of consumer health IT interventions [31].

### Considerations for Reporting Future Studies

Characterizing studies was challenging because of lack of detail and vague or incomplete descriptions in study reporting. Lack of detail was evident in the large number of articles that did not specify the technology platform used. This required an “unspecified” category for technology platform to be made. Vague or incomplete intervention descriptions were evident in our classification of cultural tailoring. Future studies should explicitly detail cultural tailoring processes and should cite feasibility studies or other evidence-based rationales to substantiate these tailoring choices. Ultimately, our ability to report frequency statistics was limited by a lack of standardized reporting methods across studies.

A consensus statement for reporting consumer health IT studies would improve the prospects for valuable meta-analyses to be conducted in the future. Consensus statements have been developed for many other types of studies, such as RCTs [133], systematic reviews [134], and Internet surveys [135]. Some of these guidelines are applicable to the consumer health IT studies reported in this paper. For example, several studies use an RCT design [96,98,136-139] and therefore may be reported using Consolidated Standards of Reporting Trials (CONSORT) guidelines [140]. However, the guidelines for CONSORT focus on study design elements and do not provide guidance for reporting aspects related to technology design. The development of a consensus statement for reporting culturally informed health IT studies, which includes both experimental and technology design elements, is important for advancing our understanding of how culture might be integrated into health information technologies. Such a consensus statement might include technology design elements such as technology functionality, user interface, content, and technology platform, as well as demographic information including country of birth, self-reported ethnicity, and user language preference.

### Limitations

Several study limitations warrant mention. Given restrictions in database access and time, a limited number of databases was chosen and additional mechanisms for hand searching were not undertaken. Furthermore, only English language articles were included. This likely contributed to selection bias and limitations in the scope of studies compiled for screening. Nonetheless, the authors used a wide range of databases from both health and computer sciences to capture a rich body of articles. Limitations were additionally faced in the classification of cultural tailoring categories. Some studies justified selecting a particular technology platform; in other cases, it was unclear whether selection of a technology platform was founded upon population-specific needs assessments or usage statistics. In these latter cases, the intervention was not considered to be culturally tailored in terms of technology platform. This approach likely led to a conservative estimate of cultural tailoring and reveals a need for more explicit descriptions of decision-making approaches used to design and develop consumer health IT interventions. Finally, the inductively derived categories were representative only of characteristics present within the studies. The scope of these categories does not give a sense of characteristics that should ideally be included but were not represented.

### Conclusions

In this study, we have characterized the growing body of consumer health IT interventions targeted toward the US Spanish-speaking Latino population. In doing so, three primary needs have been identified within this field. First, while the increase in studies targeting the Latino population in the last decade is a promising advancement, future research is needed that focuses on subpopulations previously overlooked in designing interventions within this space. For example, the Latino migrant farmworker community faces acute health conditions such as pesticide exposure, which may pose a more immediate health threat than the chronic diseases plaguing the statistical majority of the Latino demographic. Second, preliminary steps have been taken to culturally tailor consumer health IT interventions for the US Spanish-speaking Latino population; however, focus has remained predominantly on intervention content. Interdisciplinary fieldwork between the health sciences and engineering is needed to understand how to create technology culturally tailored in terms of platform, user interface, and functionality preferences. Finally, the majority of studies used intermediary measures such as knowledge retention and behavior/attitude change to evaluate technology efficacy. Given the immense financial investment and potential social benefits of consumer health IT, it is critical that research within the field engages patients long enough to begin measuring health outcomes.

## Appendix

Multimedia Appendix 1 Boolean search strings across databases.

Multimedia Appendix 2 Full-text exclusion justification breakdown.

Multimedia Appendix 3 List of excluded studies.

Multimedia Appendix 4 General study characteristics of included studies.

Multimedia Appendix 5 Participant demographics of included studies.

Multimedia Appendix 6 Intervention characteristics of included studies.

Multimedia Appendix 7 Outcome metrics, study design, and evaluation results of included studies.

## Abbreviations

CINAHL: Cumulative Index of Nursing and Allied Health Literature
IEEE: Institute of Electrical and Electronics Engineers
IT: information technology
MeSH: Medical Subject Headings
PAM: Patient Activation Measure
RCT: randomized controlled trial

## References

[1] Hibbard JH, Stockard J, Mahoney ER, Tusler M (2004). Development of the Patient Activation Measure (PAM): conceptualizing and measuring activation in patients and consumers. Health Serv Res.

[2] James J (2013). Health Affairs.

[3] Coulter A (2012). Patient engagement--what works?. J Ambul Care Manage.

[4] Ishikawa H, Yano E (2008). Patient health literacy and participation in the health-care process. Health Expect.

[5] Maurer M, Dardess P, Carman K, Frazier K, Smeeding L (2012). Guide to Patient and Family Engagement: Environmental Scan Report. AHRQ Publication No. 12-0042-EF.

[6] Dy SM, Purnell TS (2012). Key concepts relevant to quality of complex and shared decision-making in health care: a literature review. Soc Sci Med.

[7] Parsons S, Winterbottom A, Cross P, Redding D (2010). The quality of patient engagement and involvement in primary care. An Inquiry into the Quality of General Practice in England.

[8] Greene J, Hibbard JH, Sacks R, Overton V, Parrotta CD (2015). When patient activation levels change, health outcomes and costs change, too. Health Aff (Millwood).

[9] Hibbard JH, Greene J (2013). What the evidence shows about patient activation: better health outcomes and care experiences; fewer data on costs. Health Aff (Millwood).

[10] Greene J, Hibbard JH (2012). Why does patient activation matter? An examination of the relationships between patient activation and health-related outcomes. J Gen Intern Med.

[11] Mosen DM, Schmittdiel J, Hibbard J, Sobel D, Remmers C, Bellows J (2007). Is patient activation associated with outcomes of care for adults with chronic conditions?. J Ambul Care Manage.

[12] Hibbard JH, Mahoney ER, Stock R, Tusler M (2007). Do increases in patient activation result in improved self-management behaviors?. Health Serv Res.

[13] Dentzer S (2013). Rx for the 'blockbuster drug' of patient engagement. Health Aff (Millwood).

[14] Barello S, Triberti S, Graffigna G, Libreri C, Serino S, Hibbard J, Riva G (2015). eHealth for Patient Engagement: A Systematic Review. Front Psychol.

[15] Or CKL, Karsh B (2009). A systematic review of patient acceptance of consumer health information technology. J Am Med Inform Assoc.

[16] Grinter R, Siek K, Grimes A (2010). Is wellness informatics a field of human-centered health informatics?. interactions.

[17] Flaherty D, Hoffman-Goetz L, Arocha JF (2015). What is consumer health informatics? A systematic review of published definitions. Inform Health Soc Care.

[18] Gibbons M, Wilson RS, Samal L (2009). AHRQ Evidence Reports. Impact of Consumer Health Informatics Applications Internet.

[19] Keselman A, Logan R, Smith CA, Leroy G, Zeng-Treitler Q (2008). Developing informatics tools and strategies for consumer-centered health communication. J Am Med Inform Assoc.

[20] Eichner J, Dullabh P (2007). Accessible Health Information Technology (IT) for Populations with Limited Literacy: A Guide for Developers and Purchasers of Health IT Internet.

[21] Montague E, Perchonok J (2012). Health and wellness technology use by historically underserved health consumers: systematic review. J Med Internet Res.

[22] Handel MJ (2011). mHealth (mobile health)-using Apps for health and wellness. Explore (NY).

[23] López L, Grant RW (2012). Closing the gap: eliminating health care disparities among Latinos with diabetes using health information technology tools and patient navigators. J Diabetes Sci Technol.

[24] van den Berg N, Schumann M, Kraft K, Hoffmann W (2012). Telemedicine and telecare for older patients--a systematic review. Maturitas.

[25] Free C, Phillips G, Galli L, Watson L, Felix L, Edwards P, Patel V, Haines A (2013). The effectiveness of mobile-health technology-based health behaviour change or disease management interventions for health care consumers: a systematic review. PLoS Med.

[26] Gibbons MC, Wilson RF, Samal L, Lehmann CU, Dickersin K, Lehmann HP, Aboumatar H, Finkelstein J, Shelton E, Sharma R, Bass EB (2011). Consumer health informatics: results of a systematic evidence review and evidence based recommendations. Transl Behav Med.

[27] LeRouge C, Wickramasinghe N (2013). A review of user-centered design for diabetes-related consumer health informatics technologies. J Diabetes Sci Technol.

[28] Wells KJ, Vasquez-Otero C, Bredice M, Meade CD, Chaet A, Rivera MI, Arroyo G (2015). Hispanic Healthcare International. Acceptability of a Virtual Patient Educator for Hispanic Women.

[29] Sands DZ, Wald JS (2014). Transforming health care delivery through consumer engagement, health data transparency, and patient-generated health information. Yearb Med Inform.

[30] Christopher GM (2011). Use of health information technology among racial and ethnic underserved communities. Perspect Health Inf Manag.

[31] Or CKL, Tao D (2014). Does the use of consumer health information technology improve outcomes in the patient self-management of diabetes? A meta-analysis and narrative review of randomized controlled trials. Int J Med Inform.

[32] Hall AK, Cole-Lewis H, Bernhardt JM (2015). Mobile text messaging for health: a systematic review of reviews. Annu Rev Public Health.

[33] Free C, Phillips G, Galli L, Watson L, Felix L, Edwards P, Patel V, Haines A (2013). The effectiveness of mobile-health technology-based health behaviour change or disease management interventions for health care consumers: a systematic review. PLoS Med.

[34] Mignerat M, Lapointe L, Vedel I (2014). Using telecare for diabetic patients: A mixed systematic review. Health Policy and Technology.

[35] Valdez RS, Brennan PF (2015). Exploring patients' health information communication practices with social network members as a foundation for consumer health IT design. Int J Med Inform.

[36] Demiris G, Afrin LB, Speedie S, Courtney KL, Sondhi M, Vimarlund V, Lovis C, Goossen W, Lynch C (2008). Patient-centered applications: use of information technology to promote disease management and wellness. A white paper by the AMIA knowledge in motion working group. J Am Med Inform Assoc.

[37] Valdez RS, Holden RJ, Novak LL, Veinot TC (2015). Transforming consumer health informatics through a patient work framework: connecting patients to context. J Am Med Inform Assoc.

[38] Moen A, Brennan PF (2005). Health@Home: the work of health information management in the household (HIMH): implications for consumer health informatics (CHI) innovations. J Am Med Inform Assoc.

[39] Gasson S (2003). Human-Centered Vs. User-Centered Approaches to Information System Design. The Journal of Information Technology Theory and Application.

[40] De Vito Dabbs A, Myers BA, Mc Curry KR, Dunbar-Jacob J, Hawkins RP, Begey A, Dew MA (2009). User-centered design and interactive health technologies for patients. Comput Inform Nurs.

[41] Marquard JL, Zayas-Cabán T (2012). Commercial off-the-shelf consumer health informatics interventions: recommendations for their design, evaluation and redesign. J Am Med Inform Assoc.

[42] Stead WW, Lin H (2009). Computational Technology for Effective Health Care: Immediate Steps and Strategic Directions.

[43] Zayas-Cabán T, Dixon B (2010). Considerations for the design of safe and effective consumer health IT applications in the home. Qual Saf Health Care Internet.

[44] Karsh B, Weinger MB, Abbott PA, Wears RL (2010). Health information technology: fallacies and sober realities. J Am Med Inform Assoc.

[45] Forsythe DE (1996). New bottles, old wine: hidden cultural assumptions in a computerized explanation system for migraine sufferers. Med Anthropol Q.

[46] Valdez R, Gibbons M, Siegel E, Kukafka R, Brennan P (2012). Designing consumer health IT to enhance usability among different racial and ethnic groups within the United States. Health Technol.

[47] Choong Y, Salvendy G (1998). Design of icons for use by Chinese in mainland China. Interacting with Computers.

[48] Eagle PF, Marcos LR (1980). Factors in medical students' choice of psychiatry. Am J Psychiatry.

[49] Gibbons MC (2008). eHealth solutions for healthcare disparities.

[50] Koh H, Gracia N, Alvarez M (2014). Culturally and Linguistically Appropriate Services: Advancing Health with CLAS. N Engl J Med Internet.

[51] Valdez R (2012). Creating a Foundation for the Design of Culturally-Informed Health Information Technology.

[52] López L, Green AR, Tan-McGrory A, King R, Betancourt JR (2011). Bridging the digital divide in health care: the role of health information technology in addressing racial and ethnic disparities. Jt Comm J Qual Patient Saf.

[53] US Department of Health and Human Services (2011). National Partnership for Action to End Health Disparities.

[54] Cooper LA, Hill MN, Powe NR (2002). Designing and evaluating interventions to eliminate racial and ethnic disparities in health care. J Gen Intern Med.

[55] United States Census Bureau (2016). American Fact Finder.

[56] (1975). Agency for Healthcare Research and Quality.

[57] Lopez M, Gonzalez-Barrera A, Patten E (2013). Pew Research Center.

[58] DuBard A, Gizlice Z (2008). Language Spoken and Differences in Health Status, Access to Care, and Receipt of Preventive Services Among US Hispanics. Am J Public Health.

[59] Grant MJ, Booth A (2009). A typology of reviews: an analysis of 14 review types and associated methodologies. Health Info Libr J.

[60] McHugh ML (2012). Interrater reliability: the kappa statistic. Biochem Med (Zagreb).

[61] Moher D, Liberati A, Tetzlaff J, Altman DG (2009). Preferred reporting items for systematic reviews and meta-analyses: the PRISMA statement. PLoS Med.

[62] Ulrichsweb: Global serials directory.

[63] US Census Bureau Census Regions and Divisions of the United States.

[64] Trochim W, Donnelly J (2006). Research Methods Knowledge Base.

[65] Aitken M (2013). IMS Health Incorporated.

[66] Arora S, Burner E, Terp S, Nok LC, Nercisian A, Bhatt V, Menchine M (2015). Improving attendance at post-emergency department follow-up via automated text message appointment reminders: a randomized controlled trial. Acad Emerg Med.

[67] Brown SA, Duchin SP, Villagomez ET (1992). Diabetes education in a Mexican-American population: pilot testing of a research-based videotape. Diabetes Educ.

[68] Calderón JL, Bazargan M, Sangasubana N, Hays RD, Hardigan P, Baker RS (2010). A comparison of two educational methods on immigrant Latinas breast cancer knowledge and screening behaviors. J Health Care Poor Underserved.

[69] Collins TC, Dong F, Ablah E, Parra-Medina D, Cupertino P, Rogers N, Ahlers-Schmidt CR (2014). Text messaging to motivate exercise among Latino adults at risk for vascular disease: a pilot study, 2013. Prev Chronic Dis.

[70] Evans WD, Wallace JL, Snider J (2012). Pilot evaluation of the text4baby mobile health program. BMC Public Health.

[71] Frates J, Bohrer GG, Thomas D (2006). Promoting organ donation to Hispanics: the role of the media and medicine. J Health Commun.

[72] Freda MC, Damus K, Andersen HF, Brustman LE, Merkatz IR (1990). A “PROPP” for the Bronx: preterm birth prevention education in the inner city. Obstet Gynecol.

[73] Gilliam M, Tapia V, Goldstein C (2003). Increasing Contraceptive Use Among Sexually Active Latinas: Evaluation of a Self-Efficacy Enhancing Videotape. Hispanic Health Care International.

[74] King AC, Bickmore TW, Campero MI, Pruitt LA, Yin JL (2013). Employing virtual advisors in preventive care for underserved communities: results from the COMPASS study. J Health Commun.

[75] Gerber B, Lawless K, Smolin L, Brodsky I, Girotti M, Pelaez L, Eiser A (2002). Diabetes and Your Eyes: A Pilot Study on Multimedia Education for Underserved Populations. American Medical Informatics Association.

[76] Makoul G, Cameron KA, Baker DW, Francis L, Scholtens D, Wolf MS (2009). A multimedia patient education program on colorectal cancer screening increases knowledge and willingness to consider screening among Hispanic/Latino patients. Patient Educ Couns.

[77] Matthews PH, Darbisi C, Sandmann L, Galen R, Rubin D (2009). Disseminating health information and diabetes care for Latinos via electronic information kiosks. J Immigr Minor Health.

[78] McDonald DD, Gifford T, Walsh S (2011). Effect of a virtual pain coach on older adults' pain communication: a pilot study. Pain Manag Nurs.

[79] Osilla KC, D'Amico EJ, Díaz-Fuentes CM, Lara M, Watkins KE (2012). Multicultural web-based motivational interviewing for clients with a first-time DUI offense. Cultur Divers Ethnic Minor Psychol.

[80] Porter S, Chapman-Novakofski K, Scherer J (2009). Your Guide to Diet and Diabetes: web-based diabetes education tailored to Hispanics. Journal of Nutrition Education and Behavior.

[81] Quinn GP, Thomas KB, Hauser K, Rodríguez NY, Rodriguez-Snapp N (2009). Evaluation of educational materials from a social marketing campaign to promote folic acid use among Hispanic women: insight from Cuban and Puerto Rican ethnic subgroups. J Immigr Minor Health.

[82] Reuland DS, Ko LK, Fernandez A, Braswell LC, Pignone M (2012). Testing a Spanish-language colorectal cancer screening decision aid in Latinos with limited English proficiency: results from a pre-post trial and four month follow-up survey. BMC Med Inform Decis Mak.

[83] Rosas LG, Trujillo C, Camacho J, Madrigal D, Bradman A, Eskenazi B (2014). Acceptability of health information technology aimed at environmental health education in a prenatal clinic. Patient Educ Couns.

[84] Scheinmann R, Chiasson MA, Hartel D, Rosenberg TJ (2010). Evaluating a bilingual video to improve infant feeding knowledge and behavior among immigrant Latina mothers. J Community Health.

[85] Stockwell MS, Hofstetter AM, DuRivage N, Barrett A, Fernandez N, Vargas CY, Camargo S (2015). Text message reminders for second dose of influenza vaccine: a randomized controlled trial. Pediatrics.

[86] Suarez-Balcazar Y, Friesema J, Lukyanova V (2013). Culturally competent interventions to address obesity among African American and Latino children and youth. Occup Ther Health Care.

[87] Thompson DA, Joshi A, Hernandez RG, Bair-Merritt MH, Arora M, Luna R, Ellen JM (2012). Nutrition education via a touchscreen: a randomized controlled trial in Latino immigrant parents of infants and toddlers. Acad Pediatr.

[88] Valdez A, Banerjee K, Ackerson L, Fernandez M (2002). A multimedia breast cancer education intervention for low-income Latinas. J Community Health.

[89] Vaughn S (2012). Stroke and heart disease prevention education via telenovela: a focus group's evaluation. Rehabil Nurs.

[90] West AM, Bittner EA, Ortiz VE (2014). The effects of preoperative, video-assisted anesthesia education in Spanish on Spanish-speaking patients' anxiety, knowledge, and satisfaction: a pilot study. J Clin Anesth.

[91] Zyskind A, Jones K, Pomerantz K, LaFaye BA (2009). Exploring the use of computer-based patient education resources to enable diabetic patients from underserved populations to self-manage their disease. Information Services and Use.

[92] Alvaro EM, Jones SP, Robles ASM, Siegel J (2006). Hispanic organ donation: impact of a Spanish-language organ donation campaign. J Natl Med Assoc.

[93] Alvaro EM, Siegel JT, Crano WD, Dominick A (2010). A mass mediated intervention on Hispanic live kidney donation. J Health Commun.

[94] Lalonde B, Rabinowitz P, Shefsky ML, Washienko K (1997). La Esperanza del Valle: alcohol prevention novelas for Hispanic youth and their families. Health Educ Behav.

[95] Wilkin HA, Valente TW, Murphy S, Cody MJ, Huang G, Beck V (2007). Does entertainment-education work with Latinos in the United States? Identification and the effects of a telenovela breast cancer storyline. J Health Commun.

[96] Arora S, Peters AL, Burner E, Lam CN, Menchine M (2014). Trial to examine text message-based mHealth in emergency department patients with diabetes (TExT-MED): a randomized controlled trial. Ann Emerg Med.

[97] Heisler M, Choi H, Palmisano G, Mase R, Richardson C, Fagerlin A, Montori VM, Spencer M, An LC (2014). Comparison of community health worker-led diabetes medication decision-making support for low-income Latino and African American adults with diabetes using e-health tools versus print materials: a randomized, controlled trial. Ann Intern Med.

[98] Aragones A, Schwartz M, Shah N, Gany F (2010). A randomized controlled trial of a multilevel intervention to increase colorectal cancer screening among Latino immigrants in a primary care facility. J Gen Intern Med.

[99] Alcalay R, Alvarado M, Balcazar H, Newman E, Huerta E (1999). Salud para su Corazón: a community-based Latino cardiovascular disease prevention and outreach model. J Community Health.

[100] de Nuncio ML, Price SA, Tjoa T, Lashuay N, Jones MC, Elder JP (1999). Pretesting Spanish-language educational radio messages to promote timely and complete infant immunization in California. J Community Health.

[101] Steckler A, Allegrante JP, Altman D, Brown R, Burdine JN, Goodman RM, Jorgensen C (1995). Health education intervention strategies: recommendations for future research. Health Educ Q.

[102] Chen W (2001). The Relationship between Health Education and Health Promotion: A Personal Perspective. American Journal of Health Education.

[103] Peña-Purcell N (2008). Hispanics' use of Internet health information: an exploratory study. J Med Libr Assoc.

[104] Jackson K (2009). Building cultural competence: A systematic evaluation of the effectiveness of culturally sensitive interventions with ethnic minority youth. Children and Youth Services Review.

[105] Teal CR, Street RL (2009). Critical elements of culturally competent communication in the medical encounter: a review and model. Soc Sci Med.

[106] Kohli H, Huber R, Faul A (2010). Historical and Theoretical Development of Culturally Competent Social Work Practice. J Teach Soc Work.

[107] Whittemore R (2007). Culturally competent interventions for Hispanic adults with type 2 diabetes: a systematic review. J Transcult Nurs.

[108] Chin JL (2000). Culturally competent health care. Public Health Rep.

[109] Guarnaccia RO, Rodriguez O (1996). Concepts of Culture and Their Role in the Development of Culturally Competent Mental Health Services. Hispanic Journal of Behavioral Sciences.

[110] Saha S, Beach MC, Cooper LA (2008). Patient centeredness, cultural competence and healthcare quality. J Natl Med Assoc.

[111] Marcus A, Gould E (2000). Crosscurrents: cultural dimensions and global Web user-interface design. Interactions.

[112] Livingston G, Minushkin S, Cohn D (2008). Pew Research Center.

[113] Choi B, Lee I, Kim J (2006). Culturability in Mobile Data Services: A Qualitative Study of the Relationship Between Cultural Characteristics and User-Experience Attributes. International Journal of Human-Computer Interaction.

[114] Singh N, Baack D, Kundu S, Hurtado C (2008). U.S. Hispanic consumer E-commerce preferences: Expectations and attitudes toward Web content. Journal of Electronic Commerce Research.

[115] Victorson D, Banas J, Smith J, Languido L, Shen E, Gutierrez S, Cordero E, Flores L (2014). eSalud: designing and implementing culturally competent ehealth research with latino patient populations. Am J Public Health.

[116] Sachau L, Hutchinson S (2012). Trends in culturally relevant interface design features for Latino Web site users. Education Tech Research Dev.

[117] Siegel R, Miller K, Jemal A (2015). American Cancer Society.

[118] Arcury TA, Nguyen HT, Summers P, Talton JW, Holbrook LC, Walker FO, Chen H, Howard TD, Galván L, Quandt SA (2014). Lifetime and current pesticide exposure among Latino farmworkers in comparison to other Latino immigrants. Am J Ind Med.

[119] Grzywacz JG, Quandt SA, Vallejos QM, Whalley LE, Chen H, Isom S, Barr DB, Arcury TA (2010). Job demands and pesticide exposure among immigrant Latino farmworkers. J Occup Health Psychol.

[120] Sandberg J, Spears Johnson CR, Nguyen HT, Talton JW, Quandt SA, Chen H, Summers P, Arcury TA (2016). Mobile and Traditional Modes of Communication Among Male Latino Farmworkers: Implications for Health Communication and Dissemination. J Immigr Minor Health.

[121] National Council of La Raza (2014). An Inside Look at Chronic Disease and Health Care among Hispanics in the United States.

[122] Goel MS, Gracia G, Baker DW (2011). Development and pilot testing of a culturally sensitive multimedia program to improve breast cancer screening in Latina women. Patient Educ Couns.

[123] Calderón JL, Shaheen M, Hays RD, Fleming ES, Norris KC, Baker RS (2014). Improving Diabetes Health Literacy by Animation. Diabetes Educ.

[124] Byrd TL, Wilson KM, Smith JL, Coronado G, Vernon SW, Fernandez-Esquer ME, Thompson B, Ortiz M, Lairson D, Fernandez ME (2013). AMIGAS: a multicity, multicomponent cervical cancer prevention trial among Mexican American women. Cancer.

[125] Galea S, Tracy M (2007). Participation rates in epidemiologic studies. Ann Epidemiol.

[126] Meinert CL, Gilpin AK, Unalp A, Dawson C (2000). Gender representation in trials. Control Clin Trials.

[127] US Census Bureau.

[128] Bennett K, Olatosi B, Probst J South Carolina Rural Health Research Center.

[129] Ory MG, Lee SM, Mier N, Wernicke MM (2010). The science of sustaining health behavior change: the health maintenance consortium. Am J Health Behav.

[130] Eysenbach G (2005). The law of attrition. J Med Internet Res.

[131] O'Sullivan R (1991). Improving evaluation design and use through the “Evaluation Crosswalk” method. National Forum of Applied Educational Research Journal.

[132] Misra S (2012). Randomized double blind placebo control studies, the “Gold Standard” in intervention based studies. Indian J Sex Transm Dis.

[133] Schulz KF, Altman DG, Moher D (2010). [CONSORT 2010 Statement: updated guidelines for reporting parallel group randomised trials (Chinese version)]. J Chinese Integr Med.

[134] Moher D, Shamseer L, Clarke M, Ghersi D, Liberati A, Petticrew M, Shekelle P, Stewart LA, PRISMA- P (2015). Preferred reporting items for systematic review and meta-analysis protocols (PRISMA-P) 2015 statement. Syst Rev.

[135] Eysenbach G (2004). Improving the quality of Web surveys: the Checklist for Reporting Results of Internet E-Surveys (CHERRIES). J Med Internet Res.

[136] Bolin JN, Ory MG, Wilson AD, Salge L (2013). Diabetes education kiosks in a latino community. Diabetes Educ.

[137] Calles-Escandón J, Hunter JC, Langdon SE, Gómez EM, Duren-Winfield VT, Woods KF (2009). La Clínica del Pueblo: a model of collaboration between a private media broadcasting corporation and an academic medical center for health education for North Carolina Latinos. J Immigr Minor Health.

[138] Jerant A, Kravitz RL, Sohler N, Fiscella K, Romero RL, Parnes B, Tancredi DJ, Aguilar-Gaxiola S, Slee C, Dvorak S, Turner C, Hudnut A, Prieto F, Franks P (2014). Sociopsychological tailoring to address colorectal cancer screening disparities: a randomized controlled trial. Ann Fam Med.

[139] Leeman-Castillo B, Beaty B, Raghunath S, Steiner J, Bull S (2010). LUCHAR: using computer technology to battle heart disease among Latinos. Am J Public Health.

[140] Schulz KF, Altman DG, Moher D (2010). CONSORT 2010 Statement: updated guidelines for reporting parallel group randomised trials. BMJ.

