# Supplementary material for: Spanish-Language Consumer Health Information Technology Interventions: A Systematic Review
Source: J Med Internet Res. 2016 Aug 10;18(8):e214. doi: 10.2196/jmir.5794 (PMC4997005; doi:10.2196/jmir.5794)
Supplement: Multimedia Appendix 1 [file jmir_v18i8e214_app1.pdf]

Table 1. Boolean search strings across databases.

Database	Search Terms	[a]n_1=	[a]n_2=	[a]n_3=	[a]N=
**PubMed**	"Cellular Phone"[Mesh] OR "text messaging"[All Fields] OR "texting"[All Fields] OR "text message"[All Fields] OR "cell phone"[All Fields] OR "mobile phone"[All Fields] OR "mobile computing"[All Fields] OR "mhealth"[All Fields] OR "mobile health"[All Fields] OR "Tablet computer"[All Fields] OR "audio player"[All Fields] OR "Audiovisual Aids"[Mesh] OR "audiovisual"[All Fields] OR "video"[All Fields] OR "videos"[All Fields] OR "multimedia"[MeSH Terms] OR "multimedia"[All Fields] OR "Internet"[MeSH Terms] OR "internet"[All Fields] OR "blogging"[All Fields] OR "social media"[All Fields] OR "Facebook"[All Fields] OR "Twitter"[All Fields] OR "Telemedicine"[Mesh] OR "telemedicine"[All Fields] OR "Health Records, Personal"[Mesh] OR "personal health record"[All Fields] OR "personal health records"[All Fields] OR "Computer Systems"[Mesh] OR "television"[MeSH Terms] OR "television"[All Fields] OR "Medical Informatics"[Mesh] OR "health information technology"[All Fields] OR "health information technologies"[All Fields] OR "ehealth"[All Fields] OR "radio"[MeSH Terms] OR "radio"[All Fields] OR Radionovela[All Fields] OR "soap opera"[All Fields] OR "Reminder system"[All Fields] OR "Educational Technology"[MeSH Terms] OR "Computers/utilization"[MeSH Terms] OR "User-Computer Interface"[MeSH Terms] OR "computer user"[All Fields]<br><br>AND<br>"Hispanic Americans"[Majr] OR "Hispanic Americans"[All Fields] OR "Hispanic"[All Fields] OR "Hispanics"[All Fields] OR "Spanish Americans"[All Fields] OR "Latino*"[All Fields] OR "Latina*"[All Fields] OR "Spanish speaking"[All Fields]<br><br>AND<br><br>"Health Education"[Mesh] OR "Health Education"[All Fields] OR "consumer health information"[All Fields] OR "patient education"[All Fields] OR "preventive health services"[MeSH Terms] OR "Health Promotion"[MeSH Terms] OR "health promotion"[All Fields] OR "Health Care Quality, Access, and Evaluation"[Mesh] OR "Patient Compliance"[All Fields] OR "Patient Participation"[All Fields] OR "Patient Satisfaction"[All Fields] OR "Patient Preference"[All Fields]	1608	155	35	1798

**CASC**	“health education” or “consumer health information” or “patient education” or “preventive health services” or “health promotion” or “health care quality” or “health care access” or “patient compliance” or “patient participation” or “patient satisfaction” or “patient preference”) AND (“Hispanic Americans” or Hispanic or Hispanics or “Spanish Americans” or Latina* or Latino* or “Spanish speaking”) AND (“cellular phone” or “cell phone” or “text messaging” or “text message” or texting or “mobile phone” or “mobile computing” or “mhealth” or “mobile health” or “tablet computer” or “audio player” or “audiovisual aids” or audiovisual or video or videos or multimedia or internet or blogging or “social media” or Facebook or Twitter or telemedicine or “personal health record” or “personal health records” or “computer systems” or television or “medical informatics” or “health information technology” or “health information”	127	6	3	136
**Cochrane**	"health education" or "consumer health information" or "patient education" or "preventive health services" or "health promotion" or "health care quality" or "health care access" or "patient compliance" or "patient participation" or "patient satisfaction" or "patient preference" and "Hispanic Americans" or Hispanic or Hispanics or "Spanish Americans" or Latina* or Latino* or "Spanish speaking" and "cellular phone" or "cell phone" or "text messaging" or "text message" or "mobile phone" or "mobile computing" or "mhealth" or "mobile health" or "tablet computer" or "audio player" or "audiovisual aids" or audiovisual or video or videos or multimedia or internet or blogging or "social media" or Facebook or Twitter or telemedicine or "personal health record" or "personal health records" or "computer systems" or television or "medical informatics" or "health information technology" or "health information technologies" or "ehealth" or radio or radionovela or "soap opera" or "reminder system" or "educational technology" or "computer use" or "user computer interface" or "computer user" in Trials (Word variations have been searched)	87	0	0	87
**Compendex**	Same search string as in Cochrane.	5	1	0	6

**Web of Science**	[((((((((((((((((((((((((TOPIC: ("Cellular Phone") *OR* TOPIC: ("text messaging")) OR TOPIC: (texting)) OR TOPIC: ("text message"")) OR TOPIC ("cell phone")) OR TOPIC: ("mobile phone")) OR TOPIC: ("mobile computing")) OR TOPIC: ("mhealth")) OR TOPIC: ("mobile health")) OR TOPIC: ("tablet computer")) OR TOPIC ("audio player")) OR TOPIC: "audiovisual aids")) OR TOPIC: ("audiovisual")) OR TOPIC: ("video")) OR TOPIC: ("videos")) OR TOPIC: ("telemedicine")) OR TOPIC: ("personal health record")) OR TOPIC ("personal health records")) **OR** TOPIC: ("computer systems") OR TOPIC: ("television") OR TOPIC: ("medical informatics") OR TOPIC ("health information technology") OR TOPIC ("health information technologies") OR TOPIC: ("ehealth") OR TOPIC: ("radio") OR TOPIC: ("radionovela") OR TOPIC: ("soap opera") OR TOPIC: ("reminder system") OR TOPIC: ("educational technology") OR TOPIC: ("computer use") OR TOPIC: ("user-computer interface") OR TOPIC: ("computer user") **AND** TOPIC: ("Hispanic Americans") OR TOPIC: ("Hispanic") OR TOPIC: ("Hispanics") OR TOPIC: ("Spanish Americans") OR TOPIC: ("Latino") OR TOPIC: ("Latina") OR TOPIC: ("Spanish speaking")] AND TOPIC: (health education) OR TOPIC: ("consumer health information") OR TOPIC: ("patient education") OR TOPIC: ("preventative health services") OR TOPIC: ("health promotion") OR TOPIC: ("health care quality") OR TOPIC: ("health care access") OR TOPIC: ("health care evaluation") OR TOPIC ("patient compliance") OR TOPIC: ("patient participation") OR TOPIC: ("patient preference")	12	29	1	42
**CINAHL**	CINAHL<br>S1 (MH "Health Education+")<br>S2 "health education" OR "consumer health information" OR "patient education"<br>S3 ""preventive health services""S9 (MH "Hispanics")<br>S4 (MH "Health Promotion+")<br>S5 (MH "Health Services Accessibility+")<br>S6 (MH "Quality of Health Care+")<br>S7 "health promotion" OR "patient compliance" OR "patient participation" OR "patient satisfaction" OR "patient preference"<br>S8 S1 OR S2 OR S3 OR S4 OR S5 OR S6 OR S7<br>S9 (MH "Hispanics")<br>S10 "Hispanic Americans" OR "Hispanic" OR "Hispanics" OR "Spanish Americans" OR Latino* OR Latina* OR "Spanish speaking"<br>S11 S9 OR S10<br>S12 (MH "Information Science+")<br>S13 "cell phone" OR "text messaging" OR	668	30	19	717

"texting" OR "text message" OR "mobile phone" OR "mobile computing" OR "mhealh" OR "mobile health" OR "tablet computer" OR "audio player" OR "audiovisual aids" OR "audiovisual" OR "video" OR "videos" OR "multimedia" OR "multimedia" OR "internet" OR "blogging" OR "social media" OR "Facebook" OR "Twitter" OR "telemedicine" OR "personal health records" OR "personal health record" OR

S14 (MH "Informatics+")

S15 "television" OR "medical informatics" OR "health information technology" OR "health information technologies" OR "ehealth" OR "radio" OR "radionovela" OR "soap opera" OR "reminder system"

S16 (MH "Educational Technology")

S17 "computer use" OR "user computer interface" OR "computer user"

S18 S12 OR S13 OR S14 OR S15 OR S16 OR S17

S19 (MH "Health Education+")

S20 "health education" OR "consumer health information" OR "patient education"

S21 ""preventive health services""

S22 (MH "Health Promotion+")

S23 (MH "Health Services Accessibility+")

S24 (MH "Quality of Health Care+")

S25 "health promotion" OR "patient compliance" OR "patient participation" OR "patient satisfaction" OR "patient preference"

S26 S19 OR S20 OR S21 OR S22 OR S23 OR S24 OR S25

S27 (MH "Information Science+")

S28 "cell phone" OR "text messaging" OR "texting" OR "text message" OR "mobile phone" OR "mobile computing" OR "mhealh" OR "mobile health" OR "tablet computer" OR "audio player" OR "audiovisual aids" OR "audiovisual" OR "video" OR "videos" OR "multimedia" OR "multimedia" OR "internet" OR "blogging" OR "social media" OR "Facebook" OR "Twitter" OR "telemedicine" OR "personal health records" OR "personal health record" OR

S29 (MH "Informatics+")

S30 "television" OR "medical informatics" OR "health information technology" OR "health information technologies" OR "ehealth" OR "radio" OR "radionovela" OR "soap opera" OR "reminder system"

	S31 (MH "Educational Technology")<br>S32 "computer use" OR "user computer interface" OR "computer user"<br>S33 S27 OR S28 OR S29 OR S30 OR S31 OR S32<br>S34 (MH "Hispanics")<br>S35 "Hispanic Americans" OR "Hispanic" OR "Hispanics" OR "Spanish Americans" OR Latino* OR Latina* OR "Spanish speaking"<br>S36 S34 OR S35<br>S37 (MH "Health Education+")<br>S38 "health education" OR "consumer health information" OR "patient education"<br>S39 ""preventive health services""<br>S40 (MH "Health Promotion+")<br>S41 (MH "Health Services Accessibility+")<br>S42 (MH "Quality of Health Care+")<br>S43 "health promotion" OR "patient compliance" OR "patient participation" OR "patient satisfaction" OR "patient preference"<br>S44 S37 OR S38 OR S39 OR S40 OR S41 OR S42 OR S43<br>S45 S33 AND S36 AND S44				
**IEEE**	(“health education” or “consumer health information” or “patient education” or “preventive health services” or “health promotion” or “health care quality” or “health care access” or “patient compliance” or “patient participation” or “patient satisfaction” or “patient preference”) AND (“Hispanic Americans” or Hispanic or Hispanics or “Spanish Americans” or Latina* or Latino* or “Spanish speaking”) AND (“cellular phone” or “cell phone” or “text messaging” or “text message” or texting or “mobile phone” or “mobile computing” or “mhealth” or “mobile health” or “tablet computer” or “audio player” or “audiovisual aids” or audiovisual or video or videos or multimedia or internet or blogging or “social media” or Facebook or Twitter or telemedicine or “personal health record” or “personal health records” or “computer systems” or television or “medical informatics” or “health information technology” or “health information”)	0	0	0	0

[a]Where n_1 = # articles returned from August 2014 search; n_2 = # articles returned from September 2015 search. n_3 = # articles returned from June 2016 search. N= total # articles returned from database.
