# Supplementary material for: Spanish-Language Consumer Health Information Technology Interventions: A Systematic Review
Source: J Med Internet Res. 2016 Aug 10;18(8):e214. doi: 10.2196/jmir.5794 (PMC4997005; doi:10.2196/jmir.5794)
Supplement: Multimedia Appendix 2 [file jmir_v18i8e214_app2.pdf]

Table 2. Full-text exclusion justification breakdown.

Justification		# articles excluded
**R1.**	Intervention did not focus on electronic technology.	n=31
**R2.**	Program application not in Spanish.	n=49
**R3.**	Patient or caregiver is not end user.	n=15
**R4.**	Non-majority Hispanic population.	n=46
**R5.**	Results do not detail a program assessment.	n=30
**R6.**	Study is a needs assessment.	n=9
**R7.**	Intervention outside the US.	n=5
**R8.**	Duplicate article not initially identified.	n=3
**R9.**	Article published before 1990.	n=1
**R10.**	Intervention consisted only of a phone call.	n=9
