# Supplementary material for: Spanish-Language Consumer Health Information Technology Interventions: A Systematic Review
Source: J Med Internet Res. 2016 Aug 10;18(8):e214. doi: 10.2196/jmir.5794 (PMC4997005; doi:10.2196/jmir.5794)
Supplement: Multimedia Appendix 3 [file jmir_v18i8e214_app3.pdf]

List of excluded studies

(1) Acevedo A, Krueger KR, Navarro E, Ortiz F, Manly JJ, Padilla-Velez MM, et al. The Spanish translation and adaptation of the Uniform Data Set of the National Institute on Aging Alzheimer's Disease Centers. Alzheimer Dis Assoc Disord 2009 Apr-Jun;23(2):102-109.
(2) Ahlers-Schmidt CR, Ablah E, Rogers N, Cupertino P, Parra-Medina D, Dong F, et al. Low-income urban Latino parents' perceptions of immunization text reminders. Ethn Dis 2014 Spring;24(2):229-235.
(3) Ahlers-Schmidt CR, Chesser A, Brannon J, Lopez V, Shah-Haque S, Williams K, et al. "Necesita una vacuna": what Spanish-speakers want in text-message immunization reminders. J Health Care Poor Underserved 2013 Aug;24(3):1031-1041.
(4) Ahlers-Schmidt CR, Chesser AK, Paschal AM, Hart TA, Williams KS, Yaghmai B, et al. Parent opinions about use of text messaging for immunization reminders. J Med Internet Res 2012 Jun 6;14(3):e83.
(5) Albertson TE, Tharratt RS, Alsop J, Marquardt K, Heard S. Regional variations in the use and awareness of the California Poison Control System. J Toxicol Clin Toxicol 2004;42(5):625-633.
(6) Alcalay R, Alvarado M, Balcazar H, Newman E, Ortiz G. Evaluation of a community-based Latino heart disease prevention program in metropolitan Washington D.C. INT Q COMMUNITY HEALTH EDUC 1999 09;19(3):191-204.
(7) Aldridge-Gerry AA, Roesch SC, Villodas F, McCabe C, Leung QK, Da Costa M. Daily stress and alcohol consumption: modeling between-person and within-person ethnic variation in coping behavior. J Stud Alcohol Drugs 2011 Jan;72(1):125-134.
(8) Alexander J, Divin-Cosgrove C, Faner ML, O'Connell M. Increasing the knowledge base of asthmatics and their families through asthma clubs along the southwest border. J Am Acad Nurse Pract 2000 Jul;12(7):260-266.
(9) Alexander J, Kwon HT, Strecher R, Bartholomew J. Multicultural media outreach: increasing cancer information coverage in minority communities. J Cancer Educ 2013 Dec;28(4):744-747.
(10) Andersen S, Andersen P, Youngblood NE. Multimedia computerized smoking awareness education for low-literacy Hispanics. Comput Inform Nurs 2011 Feb;29(2):107-114.
(11) Anderson DR, Christison-Lagay J, Villagra V, Liu H, Dziura J. Managing the space between visits: a randomized trial of disease management for diabetes in a community health center. J Gen Intern Med 2010 Oct;25(10):1116-1122.
(12) Anderson KO, Mendoza TR, Payne R, Valero V, Palos GR, Nazario A, et al. Pain education for underserved minority cancer patients: a randomized controlled trial. J Clin Oncol 2004 Dec 15;22(24):4918-4925.
(13) Armayones M, Vilaseca MA, Cutillas J, Fabrega J, Fernandez JJ, Garcia M, et al. Guiametabolica.org: empowerment through internet tools in inherited metabolic diseases. Orphanet J Rare Dis 2012 Aug 21;7:53-1172-7-53.
(14) Arora S, Peters AL, Agy C, Menchine M. A mobile health intervention for inner city patients with poorly controlled diabetes: proof-of-concept of the TExT-MED program. Diabetes Technol Ther 2012 Jun;14(6):492-496.
(15) Arvey SR, Fernandez ME, LaRue DM, Bartholomew LK. When promotoras and technology meet: a qualitative analysis of promotoras' use of small media to increase cancer screening among South Texas Latinos. Health Educ Behav 2012 Jun;39(3):352-363.
(16) Askins MA, Sahler OJ, Sherman SA, Fairclough DL, Butler RW, Katz ER, et al. Report from a multi-institutional randomized clinical trial examining computer-assisted problem-solving skills training for English- and Spanish-speaking mothers of children with newly diagnosed cancer. J Pediatr Psychol 2009 Jun;34(5):551-563.
(17) Avis NE, Smith KW, Link CL, Goldman MB. Increasing mammography screening among women over age 50 with a videotape intervention. Prev Med 2004 Sep;39(3):498-506.
(18) Azevedo KJ, Mendoza S, Fernandez M, Haydel KF, Fujimoto M, Tirumalai EC, et al. Turn off the TV and dance! Participation in culturally tailored health interventions: implications for obesity prevention among Mexican American girls. Ethn Dis 2013 Autumn;23(4):452-461.
(19) Badger TA, Segrin C, Hepworth JT, Pasvogel A, Weihs K, Lopez AM. Telephone-delivered health education and interpersonal counseling improve quality of life for Latinas with breast cancer and their supportive partners. Psychooncology 2013;22(5):1035-1042.

(20) Baker-Ericzen MJ, Connelly CD, Hazen AL, Duenas C, Landsverk JA, Horwitz SM. A collaborative care telemedicine intervention to overcome treatment barriers for Latina women with depression during the perinatal period. Fam Syst Health 2012 Sep;30(3):224-240.
(21) Balcazar H, Alvarado M, Alcalay R, Schindeldecker M, Newman E, Huerta E, et al. Salud para su corazon: evaluating cardiovascular health outreach activities in the Latino community. MED AM 2001 2001;2(1):4-11.
(22) Bale B. Optimizing hypertension management in underserved rural populations. J Natl Med Assoc 2010 Jan;102(1):10-17.
(23) Barlow AA. Breaking through language barriers. Mark Health Serv 2001 Summer;21(2):30-31.
(24) Barnett PG, Midtling JE, Velasco AR, Romero P, O'Malley M, Clements C, et al. Educational intervention to prevent pesticide-induced illness of field workers. J Fam Pract 1984 Jul;19(1):123-125.
(25) Beach ML, Flood AB, Robinson CM, Cassells AN, Tobin JN, Greene MA, et al. Can language-concordant prevention care managers improve cancer screening rates? Cancer Epidemiol Biomarkers Prev 2007 Oct;16(10):2058-2064.
(26) Berry DC, Neal M, Hall EG, Schwartz TA, Verbiest S, Bonuck K, et al. Rationale, design, and methodology for the optimizing outcomes in women with gestational diabetes mellitus and their infants study. BMC Pregnancy Childbirth 2013 Oct 10;13:184-2393-13-184.
(27) Bertera E, M. Storytelling Slide Shows to Improve Diabetes and High Blood Pressure Knowledge and Self-Efficacy: Three-Year Results Among Community Dwelling Older African Americans. EDUC GERONTOL 2014 11;40(11):785-800.
(28) Bletzer KV. Use of ethnography in the evaluation and targeting of HIV/AIDS education among Latino farm workers. AIDS Educ Prev 1995 Apr;7(2):178-191.
(29) Boiko P, Katon W, Guerra JC, Mazzoni S. An audiotaped mental health evaluation tool for Hispanic immigrants with a range of literacy levels. J Immigr Health 2005 Jan;7(1):33-36.
(30) Borrayo EA. Where's Maria? A video to increase awareness about breast cancer and mammography screening among low-literacy Latinas. Prev Med 2004 Jul;39(1):99-110.
(31) Bowden VM, Wood FB, Warner DG, Olney CA, Olivier ER, Siegel ER. Health information Hispanic outreach in the Texas Lower Rio Grande Valley. J Med Libr Assoc 2006 Apr;94(2):180-189.
(32) Bowen GL, Richman JM, Bowen NK, Broughton A. The School Success Profile Online. Journal of Technology in Human Services 2003 01;21(1):111.
(33) Branson CE, Clemmey P, Mukherjee P. Text message reminders to improve outpatient therapy attendance among adolescents: a pilot study. Psychol Serv 2013 Aug;10(3):298-303.
(34) Brown CH, Mohr DC, Gallo CG, Mader C, Palinkas L, Wingood G, et al. A computational future for preventing HIV in minority communities: how advanced technology can improve implementation of effective programs. J Acquir Immune Defic Syndr 2013 Jun 1;63 Suppl 1:S72-84.
(35) Browner CH, Preloran M, Press NA. The effects of ethnicity, education and an informational video on pregnant women's knowledge and decisions about a prenatal diagnostic screening test. Patient Educ Couns 1996 Mar;27(2):135-146.
(36) Bryant CA, Forthofer MS, McCormack-Brown K, Alfonso ML, Quinn G. A social marketing approach to increasing breast cancer screening rates. J HEALTH EDUC 2000 2000;31(6):320-330.
(37) Bull SS, Levine DK, Black SR, Schmiege SJ, Santelli J. Social media-delivered sexual health intervention: a cluster randomized controlled trial. Am J Prev Med 2012 Nov;43(5):467-474.
(38) Buller DB, Woodall WG, Zimmerman DE, Slater MD, Heimendinger J, Waters E, et al. Randomized trial on the 5 a day, the Rio Grande Way Website, a web-based program to improve fruit and vegetable consumption in rural communities. J Health Commun 2008 Apr-May;13(3):230-249.
(39) Burner E, Menchine M, Taylor E, Arora S. Gender differences in diabetes self-management: a mixed-methods analysis of a mobile health intervention for inner-city Latino patients. J Diabetes Sci Technol 2013 Jan 1;7(1):111-118.
(40) Burns EK, Levinson AH. Reaching Spanish-speaking smokers: state-level evidence of untapped potential for QuitLine utilization. Am J Public Health 2010 03/02;100:S165-70.

(41) Burns J. Hospital, Hispanic TV station put health series on the air. Mod Healthc 1992 Mar 2;22(9):60.
(42) Cabassa LJ, Molina GB, Baron M. Depression fotonovela: development of a depression literacy tool for Latinos with limited English proficiency. Health Promot Pract 2012 Nov;13(6):747-754.
(43) Campbell NR, Ayala GX, Litrownik AJ, Slymen DJ, Zavala F, Elder JP. Evaluation of a first aid and home safety program for Hispanic migrant adolescents. Am J Prev Med 2001 May;20(4):258-265.
(44) Carroll NM, Ritzwoller DP, Stopponi MA, Johnson CC. Identifying and oversampling Hispanics by the Passel-Word surname list for enrollment in a web-based nutritional intervention. Ethn Dis 2010 Winter;20(1):15-21.
(45) Chan EC, McFall SL, Byrd TL, Mullen PD, Volk RJ, Ureda J, et al. A community-based intervention to promote informed decision making for prostate cancer screening among Hispanic American men changed knowledge and role preferences: a cluster RCT. Patient Educ Couns 2011 Aug;84(2):e44-51.
(46) Chapman E, Venkat P, Ko E, Orezzoli JP, Del Carmen M, Garner EI. Use of multimedia as an educational tool to improve human papillomavirus vaccine acceptability--a pilot study. Gynecol Oncol 2010 Aug 1;118(2):103-107.
(47) Charpentier M, Reinoso D, Dandalay L, Stelzner SM. Resident service learning project delivers health education to Hispanic community via radio. Pediatr Res 2004 APR 2004;55(4):360A-360A.
(48) Choi CS, Haynes S, Konsella L, Goodman C, Meltzer A. The Quick Health Data Online: the ultimate interactive database on recent trends in women's health. J Womens Health (Larchmt) 2007 Sep;16(7):941-958.
(49) Chong J, Moreno F. Feasibility and acceptability of clinic-based telepsychiatry for low-income Hispanic primary care patients. Telemed J E Health 2012 May;18(4):297-304.
(50) Christie J, Itzkowitz S, Lihau-Nkanza I, Castillo A, Redd W, Jandorf L. A randomized controlled trial using patient navigation to increase colonoscopy screening among low-income minorities. J Natl Med Assoc 2008 Mar;100(3):278-284.
(51) Clabots RB, Dolphin D. The multilingual videotape project: community involvement in a unique health education program. Public Health Rep 1992 Jan-Feb;107(1):75-80.
(52) Clabots RB, Dolphin D. The multilingual videotape project: community involvement in a unique health education program. Public Health Rep 1992 Jan-Feb;107(1):75-80.
(53) Colon Y. Online resources for cancer patients and social workers. Continuum 1996 Nov-Dec;16(6):6-11.
(54) Cortese J, Lustria MLA. Can tailoring increase elaboration of health messages delivered via an adaptive educational site on adolescent sexual health and decision making? J Am Soc Inf Sci Technol 2012 08;63(8):1567-1580.
(55) D'Agostino C, D'Andrea T, Nix ST, Williams CL. Increasing nutrition knowledge in preschool children: the Healthy Start project, year 1. J HEALTH EDUC 1999 1999;30(4):217-221.
(56) Dang CM, Estrada S, Bresee C, Phillips EH. Exploring potential use of internet, E-mail, and instant text messaging to promote breast health and mammogram use among immigrant Hispanic women in Los Angeles County. Am Surg 2013 Oct;79(10):997-1000.
(57) Deavenport A, Modeste N, Marshak HH, Neish C. Closing the gap in mammogram screening: an experimental intervention among low-income Hispanic women in community health clinics. Health Educ Behav 2011 Oct;38(5):452-461.
(58) Delrahim-Howlett K, Chambers CD, Clapp JD, Xu R, Duke K, Moyer RJ,3rd, et al. Web-based assessment and brief intervention for alcohol use in women of childbearing potential: a report of the primary findings. Alcohol Clin Exp Res 2011 Jul;35(7):1331-1338.
(59) DeMarco R, Norris AE. Culturally relevant HIV interventions: transcending ethnicity. J Cult Divers 2004 Summer;11(2):65-68.
(60) Derose KP, Kanouse DE, Weidmer B, Weech-Maldonado R, Garcia RE, Hays RD. Developing a Spanish-language consumer report for CAHPS health plan surveys. JOINT COMM J QUAL PATIENT SAF 2007 11;33(11):681-688.
(61) Di Noia J, Schinke SP, Pena JB, Schwinn TM. Evaluation of a brief computer-mediated intervention to reduce HIV risk among early adolescent females. J Adolesc Health 2004 Jul;35(1):62-64.

(62) Dixon-Gray LA, Mobley A, McFarlane JM, Rosenberg KD. Amor y Salud (Love and Health): a preconception health campaign for second-generation Latinas in Oregon. Am J Health Promot 2013 Jan-Feb;27(3 Suppl):S74-6.
(63) Dracup K, Moser DK, Doering LV, Guzy PM, Juarbe T. A controlled trial of cardiopulmonary resuscitation training for ethnically diverse parents of infants at high risk for cardiopulmonary arrest. Crit Care Med 2000 Sep;28(9):3289-3295.
(64) Du Bois SN, Johnson SE, Mustanski B. Examining racial and ethnic minority differences among YMSM during recruitment for an online HIV prevention intervention study. AIDS Behav 2012 Aug;16(6):1430-1435.
(65) Duggan C, Coronado G, Martinez J, Byrd TL, Carosso E, Lopez C, et al. Cervical cancer screening and adherence to follow-up among Hispanic women study protocol: a randomized controlled trial to increase the uptake of cervical cancer screening in Hispanic women. BMC Cancer 2012 May 6;12:170-2407-12-170.
(66) Dwight-Johnson M, Aisenberg E, Golinelli D, Hong S, O'Brien M, Ludman E. Telephone-based cognitive-behavioral therapy for Latino patients living in rural areas: a randomized pilot study. Psychiatr Serv 2011 Aug;62(8):936-942.
(67) Fischer HH, Moore SL, Ginosar D, Davidson AJ, Rice-Peterson CM, Durfee MJ, et al. Care by cell phone: text messaging for chronic disease management. Am J Manag Care 2012 Feb 1;18(2):e42-7.
(68) Flynn BS, Worden JK, Bunn JY, Solomon LJ, Ashikaga T, Connolly SW, et al. Mass media interventions to reduce youth smoking prevalence. Am J Prev Med 2010 Jul;39(1):53-62.
(69) Frazier M, De La Cruz N, Garces IC. Development of educational materials to improve rates of early eye care for Hispanic children. J Immigr Minor Health 2012 Aug;14(4):608-616.
(70) Frazier M, Massingale S, Bowen M, Kohler C. Engaging a community in developing an entertainment-education Spanish-language radio novella aimed at reducing chronic disease risk factors, Alabama, 2010-2011. Prev Chronic Dis 2012;9:110344.
(71) Frenn M, Malin S, Bansal N, Delgado M, Greer Y, Havice M, et al. Addressing health disparities in middle school students' nutrition and exercise. J Community Health Nurs 2003 Spring;20(1):1-14.
(72) Gao Z, Hannan P, Xiang P, Stodden DF, Valdez VE. Video game-based exercise, Latino children's physical health, and academic achievement. Am J Prev Med 2013 Mar;44(3 Suppl 3):S240-6.
(73) George S, Phillips R, McDavitt B, Adams W, Mutchler MG. The cellular generation and a new risk environment: implications for texting-based sexual health promotion interventions among minority young men who have sex with men. AMIA Annu Symp Proc 2012;2012:247-256.
(74) Gonzalez GM, Costello CR, La Tourette TR, Joyce LK, Valenzuela M. Bilingual telephone-assisted computerized speech-recognition assessment: is a voice-activated computer program a culturally and linguistically appropriate tool for screening depression in English and Spanish? Cult Divers Ment Health 1997;3(2):93-111.
(75) Gould SM, Anderson J. Using interactive multimedia nutrition education to reach low-income persons: an effectiveness evaluation. J Nutr Educ 2000 2000;32(4):204-213.
(76) Graham AL, Fang Y, Moreno JL, Streiff SL, Villegas J, Munoz RF, et al. Online advertising to reach and recruit Latino smokers to an internet cessation program: impact and costs. J Med Internet Res 2012 Aug 27;14(4):e116.
(77) Grechus M, Brown J. Comparison of individualized computer game reinforcement versus peer-interactive board game reinforcement on retention of nutrition label knowledge. J HEALTH EDUC 2000 2000;31(3):138-142.
(78) Guanipa C, Nolte LM, Lizarraga J. Using the Internet to Help Diverse Populations: A Bilingual Website. Journal of Technology in Human Services 2002 03;19(1):13.
(79) Hahn EA, Cella D. Health outcomes assessment in vulnerable populations: measurement challenges and recommendations. Arch Phys Med Rehabil 2003 Apr;84(4 Suppl 2):S35-42.
(80) Hahn EA, Cellal D, Dobrez DG, Shiomoto G, Taylor SG, Galvez AG, et al. Quality of life assessment for low literacy Latinos: a new multimedia program for self-administration. J Oncol Manag 2003 Sep-Oct;12(5):9-12.
(81) Hahn EA, Du H, Garcia SF, Choi SW, Lai JS, Victorson D, et al. Literacy-fair measurement of health-related quality of life will facilitate comparative effectiveness research in Spanish-speaking cancer outpatients. Med Care 2010 Jun;48(6 Suppl):S75-82.

(82) Hamdallah M, Vargo S, Herrera J. The VOICES/VOCES success story: effective strategies for training, technical assistance and community-based organization implementation. AIDS Educ Prev 2006 Aug;18(4 Suppl A):171-183.
(83) Harrison DF, Wambach KG, Byers JB, Imershein AW, Levine P, Maddox K, et al. AIDS knowledge and risk behaviors among culturally diverse women. AIDS Educ Prev 1991 Summer;3(2):79-89.
(84) HAYES GR, CHENG KG, HIRANO SH, TANG KP, NAGEL MS, BAKER DE. Estrellita: A Mobile Capture and Access Tool for the Support of Preterm Infants and Their Caregivers. ACM Transactions on Computer-Human Interaction (TOCHI) 2014 05;21(3):19:1-19:28.
(85) Hayes G, Patterson D, Singh M, Gravem D, Rich J, Cooper D. Supporting the transition from hospital to home for premature infants using integrated mobile computing and sensor support. Personal & Ubiquitous Computing 2011 12;15(8):871-885.
(86) Henao JC, Rodriguez J, Wilburn ST. Salsa y Salud: increasing healthy lifestyle awareness through a radio-based initiative. J Nutr Educ Behav 2006 Jul-Aug;38(4):267-268.
(87) Henderson VA, Barr KL, An LC, Guajardo C, Newhouse W, Mase R, et al. Community-based participatory research and user-centered design in a diabetes medication information and decision tool. Prog Community Health Partnersh 2013 Summer;7(2):171-184.
(88) Hofmann JN, Checkoway H, Borges O, Servin F, Fenske RA, Keifer MC. Development of a computer-based survey instrument for organophosphate and N-methyl-carbamate exposure assessment among agricultural pesticide handlers. Ann Occup Hyg 2010 Aug;54(6):640-650.
(89) Huerta EE, Weed DL. Cuidando su Salud - Spanish-language radio in preventive medicine and public health. Cancer 1998 OCT 15 1998;83(8):1805-1808.
(90) Im EO, Chang SJ, Chee W, Chee E. Attitudes of women in midlife to web-based interventions for promoting physical activity. J Telemed Telecare 2012 Oct;18(7):419-422.
(91) Im EO, Guevara E, Chee W. The pain experience of Hispanic patients with cancer in the United States. Oncol Nurs Forum 2007 Jul;34(4):861-868.
(92) Ito KE, Kalyanaraman S, Ford CA, Brown JD, Miller WC. "Let's Talk About Sex": pilot study of an interactive CD-ROM to prevent HIV/STIS in female adolescents. AIDS Educ Prev 2008 Feb;20(1):78-89.
(93) Jaganath D, Gill HK, Cohen AC, Young SD. Harnessing Online Peer Education (HOPE): integrating C-POL and social media to train peer leaders in HIV prevention. AIDS Care 2012;24(5):593-600.
(94) Jantz C, Anderson J, Gould SM. Using computer-based assessments to evaluate interactive multimedia nutrition education among low-income predominantly Hispanic participants. J Nutr Educ Behav 2002 Sep-Oct;34(5):252-260.
(95) Khanna MS, Kendall PC. Computer-assisted cognitive behavioral therapy for child anxiety: results of a randomized clinical trial. J Consult Clin Psychol 2010 Oct;78(5):737-745.
(96) Khosropour CM, Johnson BA, Ricca AV, Sullivan PS. Enhancing retention of an Internet-based cohort study of men who have sex with men (MSM) via text messaging: randomized controlled trial. J Med Internet Res 2013 Aug 27;15(8):e194.
(97) Kilanowski JF. Anticipatory guidance preferences of Latina migrant farmworker mothers. J Pediatr Health Care 2013 May-Jun;27(3):164-171.
(98) Kilanowski JF. Midwest growers' mail survey of contributors to migrant health and nutrition. J Agromedicine 2012;17(4):377-385.
(99) Kim HG, Geppert J, Quan T, Bracha Y, Lupo V, Cutts DB. Screening for postpartum depression among low-income mothers using an interactive voice response system. Matern Child Health J 2012 May;16(4):921-928.
(100) Kit Delgado M, Ginde AA, Pallin DJ, Camargo CA,Jr. Multicenter study of preferences for health education in the emergency department population. Acad Emerg Med 2010 Jun;17(6):652-658.
(101) Kralewski J, Stevens-Simon C. Does mothering a doll change teens' thoughts about pregnancy? Pediatrics 2000 Mar;105(3):E30.
(102) Kuppermann M, Norton ME, Gates E, Gregorich SE, Learman LA, Nakagawa S, et al. Computerized prenatal genetic testing decision-assisting tool: a randomized controlled trial. Obstet Gynecol 2009 Jan;113(1):53-63.
(103) Lang JM, Waterman J, Baker BL. Computeen: a randomized trial of a preventive computer and psychosocial skills curriculum for at-risk adolescents. J Prim Prev 2009 Sep;30(5):587-603.

(104) Le HN, Perry DF, Sheng X. Using the internet to screen for postpartum depression. Matern Child Health J 2009 Mar;13(2):213-221.
(105) LeBaron CW, Starnes DM, Rask KJ. The impact of reminder-recall interventions on low vaccination coverage in an inner-city population. Arch Pediatr Adolesc Med 2004 Mar;158(3):255-261.
(106) Lee C. The Role of Internet Engagement in the Health-Knowledge Gap. Journal of Broadcasting & Electronic Media 2009 07;53(3):365-382.
(107) Lehna C, Rosenberg LE, Adler-Baugh K, Epperson KM, Amrhein CA, Agular I. Family orientation to a pediatric burn ICU hospital using a DVD. Pediatr Nurs 2011 Jul-Aug;37(4):200-204.
(108) Lotfipour S, Cisneros V, Anderson CL, Roumani S, Hoonpongsimanont W, Weiss J, et al. Assessment of alcohol use patterns among spanish-speaking patients. Subst Abus 2013;34(2):155-161.
(109) Lotfipour S, Cisneros V, Chakravarthy B, Barrios C, Anderson CL, Fox JC, et al. Assessment of readiness to change and relationship to AUDIT score in a trauma population utilizing computerized alcohol screening and brief intervention. Subst Abus 2012;33(4):378-386.
(110) Ma J, Yank V, Xiao L, Lavori PW, Wilson SR, Rosas LG, et al. Translating the Diabetes Prevention Program lifestyle intervention for weight loss into primary care: a randomized trial. JAMA Intern Med 2013 Jan 28;173(2):113-121.
(111) Madrigal DS, Salvatore A, Casillas G, Casillas C, Vera I, Eskenazi B, et al. Health in My Community: Conducting and Evaluating Photo Voice as a Tool to Promote Environmental Health and Leadership Among Latino/a Youth. Progress in Community Health Partnerships-Research Education and Action 2014 FAL;8(3):317-329.
(112) Mainous AG,3rd, Diaz VA, Carnemolla M. A community intervention to decrease antibiotics used for self-medication among Latino adults. Ann Fam Med 2009 Nov-Dec;7(6):520-526.
(113) Majumdar D. P35 "Creature-101": Using a Virtual Reality, Serious Game to Promote Healthy Eating and Physical Activity Behaviors among Middle School Students. J NUTR EDUC BEHAV 2012 07/02;44(4):S38-S38.
(114) Makoul G, Francis L, Sager J, Cameron KA, Wolf MS, Baker DW. A multimedia patient education program increases colorectal cancer knowledge, risk perception, and willingness to consider screening in the Hispanic/Latino community. Journal of General Internal Medicine 2006 APR 2006;21:3-3.
(115) Markham CM, Shegog R, Leonard AD, Bui TC, Paul ME. +CLICK: harnessing web-based training to reduce secondary transmission among HIV-positive youth. AIDS Care 2009 May;21(5):622-631.
(116) Markham CM, Tortolero SR, Peskin MF, Shegog R, Thiel M, Baumler ER, et al. Sexual risk avoidance and sexual risk reduction interventions for middle school youth: a randomized controlled trial. J Adolesc Health 2012 Mar;50(3):279-288.
(117) Martin M, Holden J, Chen Z, Quinlan K. Child passenger safety for inner-city Latinos: new approaches from the community. Inj Prev 2006 Apr;12(2):99-104.
(118) Mayor A, M., Fernández D, M., Colón H, M., Thomas J, C., Miranda C, Hunter-Mellado R. Hepatitis-C Multimedia Prevention Program in Poor Hispanic HIV-Infected Injecting drug users: Six Months after Intervention. J HEALTH CARE POOR UNDERSERV 2013 11/02;24(4):29-37.
(119) McConnochie KM, Wood NE, Herendeen NE, ten Hoopen CB, Roghmann KJ. Telemedicine in urban and suburban childcare and elementary schools lightens family burdens. Telemed J E Health 2010 Jun;16(5):533-542.
(120) Miner MH, Coleman E, Center BA, Ross M, Rosser BR. The compulsive sexual behavior inventory: psychometric properties. Arch Sex Behav 2007 Aug;36(4):579-587.
(121) Moore M, Bias RG, Prentice K, Fletcher R, Vaughn T. Web usability testing with a Hispanic medically underserved population. J Med Libr Assoc 2009 Apr;97(2):114-121.
(122) Mudd GT. The development of a Spanish language instrument to measure genetic knowledge of diabetes mellitus type 2. J Transcult Nurs 2011 Apr;22(2):148-156.
(123) Mull LD, Engel LS, Outterson B, Zahm SH. National farmworker database: establishing a farmworker cohort for epidemiologic research. Am J Ind Med 2001 Nov;40(5):612-618.
(124) Munoz RF, Barrera AZ, Delucchi K, Penilla C, Torres LD, Perez-Stable EJ. International Spanish/English Internet smoking cessation trial yields 20% abstinence rates at 1 year. Nicotine Tob Res 2009 Sep;11(9):1025-1034.

(125) Murphy MK, Bijur PE, Rosenbloom D, Bernstein SL, Gallagher EJ. Feasibility of a computer-assisted alcohol SBIRT program in an urban emergency department: patient and research staff perspectives. Addict Sci Clin Pract 2013 Jan 16;8:2-0640-8-2.
(126) Mussulman L, Ellerbeck EF, Cupertino AP, Preacher KJ, Spaulding R, Catley D, et al. Design and participant characteristics of a randomized-controlled trial of telemedicine for smoking cessation among rural smokers. Contemporary Clinical Trials 2014 JUL;38(2):173-181.
(127) Nan X, Madden K. HPV Vaccine Information in the Blogosphere: How Positive and Negative Blogs Influence Vaccine-Related Risk Perceptions, Attitudes, and Behavioral Intentions. Health Commun 2012 11;27(7):829-836.
(128) Nguyen KD, Hara B, Chlebowski RT. Utility of two cancer organization websites for a multiethnic, public hospital oncology population: comparative cross-sectional survey. J Med Internet Res 2005 Jul 1;7(3):e28.
(129) Nicklas TA, Goh ET, Goodell LS, Acuff DS, Reiher R, Buday R, et al. Impact of commercials on food preferences of low-income, minority preschoolers. J Nutr Educ Behav 2011 Jan-Feb;43(1):35-41.
(130) Norris AE, Hughes C, Hecht M, Peragallo N, Nickerson D. Randomized trial of a peer resistance skill-building game for Hispanic early adolescent girls. Nurs Res 2013 Jan-Feb;62(1):25-35.
(131) O'Donnell CR, O'Donnell L, San Doval A, Duran R, Labes K. Reductions in STD infections subsequent to an STD clinic visit. Using video-based patient education to supplement provider interactions. Sex Transm Dis 1998 Mar;25(3):161-168.
(132) O'Donnell L, San Doval A, Duran R, O'Donnell CR. The effectiveness of video-based interventions in promoting condom acquisition among STD clinic patients. Sex Transm Dis 1995 Mar-Apr;22(2):97-103.
(133) O'Donnell L, San Doval A, Vornfett R, DeJong W. Reducing AIDS and other STDs among inner-city Hispanics: the use of qualitative research in the development of video-based patient education. AIDS Educ Prev 1994 Apr;6(2):140-153.
(134) O'Donnell LN, Doval AS, Duran R, O'Donnell C. Video-based sexually transmitted disease patient education: its impact on condom acquisition. Am J Public Health 1995 Jun;85(6):817-822.
(135) Owusu-Edusei K,Jr, Doshi SR. Assessing spatial gaps in sexually transmissible infection services and morbidity: an illustration with Texas county-level data from 2007. Sex Health 2012 Sep;9(4):334-340.
(136) Paiva A, L., Lipschitz J, M., Fernandez A, C., Redding C, A., Prochaska J, O. Evaluation of the Acceptability and Feasibility of a Computer-Tailored Intervention to Increase Human Papillomavirus Vaccination Among Young Adult Women. J Am Coll Health 2014;62(1):32-38.
(137) Palen LA, Ashley OS, Gard JC, Kan ML, Davis KC, Evans WD. Effects of media campaign messages targeting parents on adolescent sexual beliefs: a randomized controlled trial with a national sample. Fam Community Health 2011 Jan-Mar;34(1):28-38.
(138) Pendleton BF, Labuda Schrop S, Ritter C, Kinion ES, McCord G, Cray JJ, et al. Underserved patients' choice of kiosk-based preventive health information. Fam Med 2010 Jul-Aug;42(7):488-495.
(139) Piette JD, Marinec N, Gallegos-Cabriales EC, Gutierrez-Valverde JM, Rodriguez-Saldana J, Mendoz-Alevares M, et al. Spanish-speaking patients' engagement in interactive voice response (IVR) support calls for chronic disease self-management: data from three countries. J Telemed Telecare 2013 Feb;19(2):89-94.
(140) Piette JD, Weinberger M, McPhee SJ. The effect of automated calls with telephone nurse follow-up on patient-centered outcomes of diabetes care: a randomized, controlled trial. Med Care 2000 Feb;38(2):218-230.
(141) Pinzon-Perez H, Perez M, Torres V, Krenz V. A qualitative study about cervical cancer screening among Latinas living in a rural area of California: lessons for health educators. AM J HEALTH EDUC 2005 2005;36(4):228-236.
(142) Porter SC, Forbes P, Feldman HA, Goldmann DA. Impact of patient-centered decision support on quality of asthma care in the emergency department. Pediatrics 2006 Jan;117(1):e33-42.
(143) Quinones AR, Ramsey K, Newsom JT, Dorr DA. Racial and ethnic differences in clinical outcome trajectories for care managed patients. Med Care 2014 Nov;52(11):998-1005.

(144) Quintiliani LM, DeBiasse MA, Branco JM, Bhosrekar SG, Rorie JA, Bowen DJ. Enhancing physical and social environments to reduce obesity among public housing residents: rationale, trial design, and baseline data for the Healthy Families study. Contemp Clin Trials 2014 Nov;39(2):201-210.
(145) Quirk ME, Godkin MA, Schwenzfeier E. Evaluation of two AIDS prevention interventions for inner-city adolescent and young adult women. Am J Prev Med 1993 Jan-Feb;9(1):21-26.
(146) Ralston JD, Silverberg MJ, Grothaus L, Leyden WA, Ross T, Stewart C, et al. Use of web-based shared medical records among patients with HIV. Am J Manag Care 2013 Apr 1;19(4):e114-24.
(147) Ramirez AG, Gallion KJ, Espinoza R, Chalela P. Developing a media- and school-based program for substance abuse prevention among Hispanic youth: a case study of Mirame!/Look at Me! Nicotine Tob Res 1999;1 Suppl 1:S99-104.
(148) Reininger B, Mecca LP, Stine KM, Schultz K, Ling L, Halpern D, et al. A Type 2 Diabetes Prevention Website for African Americans, Caucasians, and Mexican Americans: Formative Evaluation. Journal of Medical Internet Research 2013 07;15(7):1-1.
(149) Reznik M, Sharif I, Ozuah PO. Use of interactive videoconferencing to deliver asthma education to inner-city immigrants. J Telemed Telecare 2004;10(2):118-120.
(150) Richter DL, Greaney ML, McKeown RE, Cornell CE, Littleton MA, Pulley L, et al. Developing a video intervention to model effective patient-physician communication and health-related decision-making skills for a multiethnic audience. J Am Med Womens Assoc 2001 Fall;56(4):174-6, 196.
(151) Riegel B, Carlson B, Glaser D, Romero T. Randomized controlled trial of telephone case management in Hispanics of Mexican origin with heart failure. J Card Fail 2006 Apr;12(3):211-219.
(152) Robbins B, Rausch KJ, Garcia RI, Prestwood KM. Multicultural medication adherence: a comparative study. J Gerontol Nurs 2004 Jul;30(7):25-32.
(153) Robinson JK, Guevara Y, Gaber R, Clayman ML, Kwasny MJ, Friedewald JJ, et al. Efficacy of a sun protection workbook for kidney transplant recipients: a randomized controlled trial of a culturally sensitive educational intervention. Am J Transplant 2014 Dec;14(12):2821-2829.
(154) Rotheram-Borus MJ, Piacentini J, Cantwell C, Belin TR, Song J. The 18-month impact of an emergency room intervention for adolescent female suicide attempters. J Consult Clin Psychol 2000 Dec;68(6):1081-1093.
(155) Roye C, Perlmutter Silverman P, Krauss B. A brief, low-cost, theory-based intervention to promote dual method use by black and Latina female adolescents: a randomized clinical trial. Health Educ Behav 2007 Aug;34(4):608-621.
(156) Sanchez JP, Guilliames C, Sanchez NF, Calderon Y, Burton WB. Video tool to promote knowledge of syphilis among black and Hispanic men recruited from clinical and non-clinical settings. J Community Health 2010 Jun;35(3):220-228.
(157) Sanchez JP, Kaltwassar S, McClellan M, Burton WB, Blank A, Calderon Y. Educational video tool to increase syphilis knowledge among black and Hispanic male patients. J Health Care Poor Underserved 2010 Feb;21(1):371-385.
(158) Saul J, Moore J, Murphy ST, Miller LC. Relationship violence and women's reactions to male- and female-controlled HIV prevention methods. AIDS Behav 2004 Jun;8(2):207-214.
(159) Schinke SP, Fang L, Cole KC, Cohen-Cutler S. Preventing substance use among Black and Hispanic adolescent girls: results from a computer-delivered, mother-daughter intervention approach. Subst Use Misuse 2011;46(1):35-45.
(160) Schinke SP, Orlandi MA, Schilling RF, Parms C. Feasibility of interactive videodisc technology to teach minority youth about preventing HIV infection. Public Health Rep 1992 May-Jun;107(3):323-330.
(161) Scholer SJ, Hudnut-Beumler J, Dietrich MS. The effect of physician--parent discussions and a brief intervention on caregivers' plan to discipline: is it time for a new approach? Clin Pediatr (Phila) 2011 Aug;50(8):712-719.
(162) Scholer SJ, Hudnut-Beumler J, Dietrich MS. A brief primary care intervention helps parents develop plans to discipline. Pediatrics 2010 Feb;125(2):e242-9.
(163) Schroy PC,3rd, Emmons K, Peters E, Glick JT, Robinson PA, Lydotes MA, et al. The impact of a novel computer-based decision aid on shared decision making for colorectal cancer screening: a randomized trial. Med Decis Making 2011 Jan-Feb;31(1):93-107.

(164) Shea S, Basch CE, Wechsler H, Lantigua R. The Washington Heights-Inwood Healthy Heart Program: a 6-year report from a disadvantaged urban setting. Am J Public Health 1996 Feb;86(2):166-171.
(165) Stockwell MS, Kharbanda EO, Martinez RA, Vargas CY, Vawdrey DK, Camargo S. Effect of a text messaging intervention on influenza vaccination in an urban, low-income pediatric and adolescent population: a randomized controlled trial. JAMA 2012 Apr 25;307(16):1702-1708.
(166) Sussman S, Parker VC, Lopes C, Crippens DL, Elder P, Scholl D. Empirical development of brief smoking prevention videotapes which target African-American adolescents. Int J Addict 1995 Jul;30(9):1141-1164.
(167) Sweat M, O'Donnell C, O'Donnell L. Cost-effectiveness of a brief video-based HIV intervention for African American and Latino sexually transmitted disease clinic clients. AIDS 2001 Apr 13;15(6):781-787.
(168) Sweeney MA, Gulino C. Interactive video in health care: blending patient care, computer technology, and research results. J Biocommun 1988 Fall;15(4):6-11.
(169) Swendeman D, Ramanathan N, Baetscher L, Medich M, Scheffler A, Comulada WS, et al. Smartphone self-monitoring to support self-management among people living with HIV: perceived benefits and theory of change from a mixed-methods randomized pilot study. J Acquir Immune Defic Syndr 2015 May 1;69 Suppl 1:S80-91.
(170) Szilagyi PG, Humiston SG, Gallivan S, Albertin C, Sandler M, Blumkin A. Effectiveness of a citywide patient immunization navigator program on improving adolescent immunizations and preventive care visit rates. Arch Pediatr Adolesc Med 2011 Jun;165(6):547-553.
(171) Tanke ED, Martinez CM, Leirer VO. Use of automated reminders for tuberculin skin test return. Am J Prev Med 1997 May-Jun;13(3):189-192.
(172) Thompson DA, Joshi A, Hernandez RG, Jennings JM, Arora M, Ellen JM. Interactive nutrition education via a touchscreen: is this technology well received by low-income Spanish-speaking parents? Technol Health Care 2012;20(3):195-203.
(173) Uebelacker LA, Marootian BA, Tigue P, Haggarty R, Primack JM, Miller IW. Telephone depression care management for Latino Medicaid health plan members: a pilot randomized controlled trial. J Nerv Ment Dis 2011 Sep;199(9):678-683.
(174) Unger JB, Molina GB, Baron M. Evaluation of Sweet Temptations, a fotonovela for diabetes education. HISPANIC HEALTH CARE INT 2009 09;7(3):145-152.
(175) Valdez A, Banerjee K, Fernandez M, Ackerson L. Impact of a multimedia breast cancer education intervention on use of mammography by low-income Latinas. J Cancer Educ 2001 Winter;16(4):221-224.
(176) Valladares AF, Aebersold M, Tschannen D, Villarruel AM. Preparing facilitators from community-based organizations for evidence-based intervention training in Second Life. J Med Internet Res 2014 Sep 30;16(9):e220.
(177) Valle R, Yamada A, Matiella AC. Fotonovelas: a health literacy tool for educating Latino older adults about dementia. Clin Gerontol 2006 09;30(1):71-88.
(178) Villarruel AM, Aebersold M, Valladares AF, Yeagley E, Tschannen D. Avatars travel for free: virtual access to evidence-based intervention training and capacity building. AIDS Educ Prev 2014 Oct;26(5):445-458.
(179) Natalia Andrea Villegas Rodriguez. Developing and piloting an internet based STI and HIV prevention intervention among young Chilean womenUniversity of Miami; 2012.
(180) Waterman AD, Robbins ML, Paiva AL, Peipert JD, Kynard-Amerson CS, Goalby CJ, et al. Your Path to Transplant: a randomized controlled trial of a tailored computer education intervention to increase living donor kidney transplant. Bmc Nephrology 2014 OCT 14;15:166.
(181) Western Institute of Nursing. Telephone education and counseling: Latina breast cancer survivors and support partners. Commun Nurs Res 2012 2012;45:245-245.
(182) Wilhelm S, Aguirre T. Feasibility of a Bilingual, Interactive, Computer-Based Breastfeeding Support Program for Rural Hispanic Women. JOGNN 2015 06/02;44:S60-S60.
(183) Wofford JL, Campos CL, Johnson DA, Brown MT. Providing a Spanish interpreter using low-cost videoconferencing in a community health centre: a pilot study using tablet computers. Inform Prim Care 2012;20(2):141-146.
(184) Yancey AK, Tanjasiri SP, Klein M, Tunder J. Increased cancer screening behavior in women of color by culturally sensitive video exposure. Prev Med 1995 Mar;24(2):142-148.

(185) Yancey AK, Walden L. Stimulating cancer screening among Latinas and African-American women. A community case study. J Cancer Educ 1994;9(1):46-52.
(186) Yellen EA, Ricard R. The effect of a preadmission videotape on patient satisfaction. AORN J 2005 Apr;81(4):831-42, 845.
(187) Yeo G. Outcomes and challenges of Familias Saludables: a rural latino chronic disease screening and management project. HISPANIC HEALTH CARE INT 2011 09;9(3):137-143.
(188) Yost KJ, Webster K, Baker DW, Choi SW, Bode RK, Hahn EA. Bilingual health literacy assessment using the Talking Touchscreen/la Pantalla Parlanchina: Development and pilot testing. Patient Educ Couns 2009 Jun;75(3):295-301.
(189) Young SD, Holloway I, Jaganath D, Rice E, Westmoreland D, Coates T. Project HOPE: online social network changes in an HIV prevention randomized controlled trial for African American and Latino men who have sex with men. Am J Public Health 2014 Sep;104(9):1707-1712.
(190) Young SD, Jaganath D. Feasibility of using social networking technologies for health research among men who have sex with men: a mixed methods study. Am J Mens Health 2014 Jan;8(1):6-14.
(191) Young SD, Jaganath D. Online social networking for HIV education and prevention: a mixed-methods analysis. Sex Transm Dis 2013 Feb;40(2):162-167.
(192) Young SD, Jaganath D. Feasibility of Using Social Networking Technologies for Health Research Among Men Who Have Sex With Men: A Mixed Methods Study. American Journal of Mens Health 2014 JAN;8(1):6-14.
(193) Yturri-Byrd K, Glazer-Waldman H, Hedl J, J., Bernardez S, Grover R. Teaching teenagers about AIDS. J AM ACAD PHYSICIAN ASSIST 1992 06;5(6):432-437.
(194) Yturri-Byrd K, Glazer-Waldman H, Jr HJ, Bernardez S, Grover R. Teaching teenagers about AIDS. J AM ACAD PHYSICIAN ASSIST 1992 1992;5(6):432-437 6p.
(195) Zabinski MF, Wilfley DE, Calfas KJ, Winzelberg AJ, Taylor CB. An interactive psychoeducational intervention for women at risk of developing an eating disorder. J Consult Clin Psychol 2004 Oct;72(5):914-919.
(196) Zarcadoolas C, Vaughon WL, Czaja SJ, Levy J, Rockoff ML. Consumers' perceptions of patient-accessible electronic medical records. J Med Internet Res 2013 Aug 26;15(8):e168.
(197) Zimmerman DE, Akerelrea CA, Buller DB, Hau B, Leblanc M. Integrating usability testing into the development of a 5 a day nutrition Website for at-risk populations in the American Southwest. J HEALTH PSYCHOL 2003;8(1):119-134.
(198) Zimmers E, Privette G, Lowe RH, Chappa F. Increasing use of the female condom through video instruction. Percept Mot Skills 1999 Jun;88(3 Pt 2):1071-1077.
