# Supplementary material for: Spanish-Language Consumer Health Information Technology Interventions: A Systematic Review
Source: J Med Internet Res. 2016 Aug 10;18(8):e214. doi: 10.2196/jmir.5794 (PMC4997005; doi:10.2196/jmir.5794)
Supplement: Multimedia Appendix 4 [file jmir_v18i8e214_app4.pdf]

Table 3. General study characteristics of included studies.

Author, Year	Full title	Journal Domain	Technology Platform	Health Concern	Sample Population Size
Alcalay, R., 1999	Salud para su Corazon: a community-based Latino cardiovascular disease prevention and outreach model	Medical Sciences; Public Health and Safety	television, radio (telenovela public service announcements)	cardiovascular disease	N=672
Alvaro, E., 2006	Hispanic organ donation: impact of a Spanish-language organ donation campaign	National Medical Association	television, radio (media campaign)	organ donation	N=2400
Alvaro, E., 2010	A mass mediated intervention on Hispanic live kidney donation	Medical Sciences; Communication	television, radio (media campaign)	organ donation	N=1648
Aragones, A., 2010	A Randomized Controlled Trial of a Multilevel Intervention to Increase Colorectal Cancer Screening among Latino Immigrants in a Primary Care Facility	Medical Sciences - Internal Medicine	DVD	cancer - colorectal cancer screenings	N=65
Arora, S., 2014	Trial to examine text message-based mHealth in emergency department patients with diabetes (TExT-MED): a randomized controlled trial	Medical Sciences - Orthopedics and Traumatology	cellphone - text message	diabetes	N=128
Arora, S., 2015	Improving attendance at post-emergency department follow-up via automated text message appointment reminders: a randomized controlled trial	Medical Sciences - Orthopedics and Traumatology	cellphone - text message	appointment reminder - post-emergency department follow-up	N=374

Bolin, J., 2013	Diabetes education kiosks in a Latino community	Medical Sciences - Endocrinology	kiosk	diabetes	N=179
Brown, S., 1992	Diabetes education in a Mexican-American population: pilot testing of a research-based videotape	Medical Sciences- Endocrinology	VCR (videotape)	Diabetes	N=30
Byrd, T., 2013 [100]	AMIGAS: a multicity, multicomponent cervical cancer prevention trial among Mexican American women	Medical Sciences - Oncology	unspecified	cancer - cervical cancer	N=613
Calderón, J., 2010	A comparison of two educational methods on immigrant Latinas breast cancer knowledge and screening behaviors	Medical Sciences; Social Services and Welfare	unspecified	cancer - mammography and breast self exam	N=350
Calderón, J., 2014	Improving Diabetes Health Literacy by Animation	Medical Sciences - Endocrinology	unspecified	diabetes	N=240
Calles-Escandón, J., 2009	La Clinica del Pueblo: a model of collaboration between a private media broadcasting corporation and an academic medical center for health education for North Carolina Latinos	Medical Sciences; Ethnic Interests	radio	childhood health, adult health, safety, and healthcare utilization	N=298
Collins, T., 2014	Text messaging to motivate exercise among Latino adults at risk for vascular disease: a pilot study, 2013	Medical Sciences	cellphone - text message	physical activity	N=11
de Nuncio, M., 1999	Pretesting Spanish-language educational radio messages to promote timely and complete infant immunization in California	Medical Sciences; Public Health and Safety	radio	child health - infant immunization	N=64

Evans, W.D., 2012	Pilot evaluation of the text4baby mobile health program	Public Health and Safety	cellphone - text message	maternal and infant health - prenatal and postpartum physical and mental health	N=123
Frates, J., 2006	Promoting organ donation to Hispanics: the role of the media and medicine	Medical Sciences; Communication	television and radio	organ donation	N=500
Freda, M.C., 1990	A “PROPP” for the Bronx: preterm birth prevention education in the inner city	Medical Sciences- Obstetrics and Gynecology	VCR (videotape)	Maternal and infant health – preterm birth prevention	N=615
Gerber, B., 2005	Diabetes and Your Eyes: a Pilot Study on Multimedia Education for Underserved Populations	Education - Teaching Methods and Curriculum; Technology: Comprehensive Works	kiosk	diabetes - diabetic eye care	N=56
Gilliam, M., 2003	Increasing contraceptive use among sexually active Latinas: evaluation of a self-efficacy enhancing videotape	Medical Sciences - Nurses and Nursing; Ethnic Interests	VCR (videotape)	birth control - contraception use	N=13
Goel, M.S., 2011	Development and pilot testing of a culturally sensitive multimedia program to improve breast cancer screening in Latina women	Public Health and Safety	unspecified	cancer - breast cancer screening	N=91
Heisler, M., 2014	Comparison of community health worker-led diabetes medication decision-making support for low-income Latino and African American adults with diabetes using e-health tools versus print materials: a randomized, controlled trial	Medical Sciences - Orthopedics and Traumatology	tablet	diabetes	N=188

Jerant, A., 2014	Sociopsychological Tailoring to Address Colorectal Cancer Screening Disparities: A Randomized Controlled Trial	Medical Sciences	computer	cancer - colorectal cancer screening	N=1164
King, A., 2013	Employing Virtual Advisors in Preventive Care for Underserved Communities: Results From the COMPASS Study.	Medical Sciences; Communication	computer (embodied conversational agent)	physical activity	N=40
Lalonde, B., 1997	La Esperanza del Valle: alcohol prevention novelas for Hispanic youth and their families	Education - Adult Education; Public Health and Safety	radio, television	child health - youth substance abuse prevention (alcohol, tobacco, and drugs)	Street Interview: N=648 High School Telenovela Interviews: N=646
Leeman-Castillo, B., 2010	LUCHAR: using computer technology to battle heart disease among Latinos	Public Health and Safety	kiosk	cardiovascular disease	N=299
Makoul, G., 2009	A multimedia patient education program on colorectal cancer screening increases knowledge and willingness to consider screening among Hispanic/Latino patients	Public Health and Safety	kiosk	cancer - colorectal cancer education and screening	N=270
Matthews, P., 2009	Disseminating health information and diabetes care for Latinos via electronic information kiosks	Medical Sciences; Ethnic Interests	kiosk	diabetes	N=24

McDonald, D., 2012	The effect of a Spanish virtual pain coach for older adults: a pilot study	Medical Sciences - Psychiatry and Neurology; Medical Sciences - Anesthesiology	computer (virtual pain coach video)	pain communication for osteoporosis	N=18
Osilla, K., 2012	Multicultural web-based motivational interviewing for clients with a first-time DUI offense	Psychology; Sociology	computer	DUI recidivism	N=48
Porter, S., 2009	Your Guide to Diet and Diabetes: web-based diabetes education tailored to Hispanics	Nutrition and Dietetics	computer	diabetes	N=9
Quinn, G., 2009	Evaluation of educational materials from a social marketing campaign to promote folic acid use among Hispanic women: insight from Cuban and Puerto Rican ethnic subgroups	Medical Sciences; Ethnic Interests	unspecified (videonovela)	maternal health - folic acid use	N=74
Reuland, D., 2012	Testing a Spanish-language colorectal cancer screening decision aid in Latinos with limited English proficiency: results from a pre-post trial and four month follow-up survey	Medical Sciences - Computer Applications	computer	cancer - colorectal cancer education and screening	N=80
Rosas, L., 2014	Acceptability of health information technology aimed at environmental health education in a prenatal Clinic	Public Health and Safety	kiosk	maternal and infant health - prenatal environmental	N=152

Scheinmann, R., 2010	Evaluating a bilingual video to improve infant feeding knowledge and behavior among immigrant Latina mothers	Medical Sciences; Public Health and Safety	DVD	child health - infant feeding	N=272
Stockwell, M.S., 2015	Text message reminders for second dose of influenza vaccine: a randomized controlled trial	Medical Sciences - Pediatrics	cellphone - text message	immunization - influenza vaccination reminder	N=660
Suarez, L., 1993	Use of peer role models to increase Pap smear and mammogram screening in Mexican-American and black women	Medical Sciences; Public Health and Safety	television, radio	breast and cervical cancer screening	N=209
Thompson, D.A., 2012	Nutrition education via a touchscreen: a randomized controlled trial in Latino immigrant parents of infants and toddlers	Medical Sciences - Pediatrics	computer	child health - pediatric nutrition	N=160
Valdez, A., 2002	A multimedia breast cancer education intervention for low-income Latinas	Medical Sciences; Public Health and Safety	kiosk	breast cancer	N=1197
Vaughn. S., 2012	Stroke and heart disease prevention education via telenovela: a focus group's evaluation	Medical Sciences - Physical Medicine and Rehabilitation; Medical Sciences - Nurses and Nursing	television (telenovela)	cardiovascular disease and stroke prevention	N=12
West, A., 2014	The effects of preoperative, video-assisted anesthesia education in Spanish on Spanish-speaking patients' anxiety, knowledge, and satisfaction: a pilot study	Medical Sciences - Anesthesiology	tablet	anesthesia	N=20

Wilkin, H.A., 2007	Does entertainment-education work with Latinos in the United States? Identification and the effects of a telenovela breast cancer storyline	Medical Sciences; Communication	television (telenovela)	cancer - breast cancer	N=2516
Zyskind, A., 2009	Exploring the use of computer based patient education resources to enable diabetic patients from underserved populations to self-manage their disease	Library and Information Sciences; Computers - Information science and information theory	computer (Internet video through MedlinePlus)	diabetes	N=108
