# Supplementary material for: Spanish-Language Consumer Health Information Technology Interventions: A Systematic Review
Source: J Med Internet Res. 2016 Aug 10;18(8):e214. doi: 10.2196/jmir.5794 (PMC4997005; doi:10.2196/jmir.5794)
Supplement: Multimedia Appendix 5 [file jmir_v18i8e214_app5.pdf]

Table 4. Participant demographics of included studies.

Author, Year	Female/Male	Ethnicity	Age (mean unless otherwise specified)	% Hispanic	State	Intervention location: urban/rural
Alcalay, R., 1999	one half female; one half male	DNS	50% between 18-34; 50% between 35-54	N=672, 100%	Virginia	urban
Alvaro, E., 2006	female (n=1572, 65.5%); male (n=1068, 44.5%)	Mexican, study location 1: pre, post test (35.7%, 30.6%) study location 2: pre-post survey: (35.7%, 30.6%)	40.4	N=2400, 100%	Arizona	urban
Alvaro, E., 2010	female (n=1055, 64%); male (n=593, 36%)	DNS	47.4	N=1648, 100%	Arizona	urban
Aragones, A., 2010	female (n=33, 51%); male (n=32, 49%)	DNS	58.3	N=65, 100%	New York	urban

Arora, S., 2014	female (n=82, 64%); male (n=46, 36%)	DNS	50.7	n=112, 87%	California	urban
Arora, S., 2015	female (n=196, 52.4%); male (n=178, 47.6%)	DNS	45.6	n=266, 71.1%	California	urban
Bolin, J., 2013	female (n=115, 63.7%); male (n=64, 30.8%); no response (n=9, 5.5%)	"women of Mexican origin"	<18: n=18, 9.89%<br>19-35: n=28, 15.38%<br>36-49: n=71, 39.01%<br>50-64: n=44, 24.18%<br>65+: n=10, 5.49%<br>DNS: n=11, 6.04%	n=120, 65.9%	Texas	urban
Brown, S., 1992	Female (n= 21, 70%) Male (n=9, 30%)	DNS	59	N=30, 100%	Texas	rural
Byrd, T., 2013	female (N=613, 100%)	"nearly all of Mexican heritage"	21+	N=613, 100%	Texas; Washington	urban and rural

Calderón, J., 2010	female (N=350, 100%)	Mexico (50%); El Salvador (24%), Guatemala (8%), Honduras (8%); Costa Rica (9%), Cuba (9%); other Latin American countries (8%)	30-39: n=141, 40.2%<br>40-49: n=108, 30.8%<br>50-59: n=68, 19.4%<br>60+: n= 33, 9.4%	N=350, 100%	California	urban
Calderón, J., 2014	female (n=196, 81.7%);<br>male (n=44, 18.3%)	DNS	18-39: n=49, 20.7%<br>40-60: n=139, 88.6%<br>60+: n=49, 20.7%	majority Hispanic	California	urban
Calles-Escandón, J., 2009	female (n=179, 60%);<br>male (n=119, 40%)	Mexico (37.5%); US (47.5%); Other (15%)	<38: n= 209, 70%	N=298, 100%	North Carolina	urban
Collins, T., 2014	female (n=10, 90.9%);<br>male (n=1, 9.1%)	DNS	51.5	N=11, 100%	Kansas	urban
de Nuncio, M., 1999	female (n=61, 95%);<br>male (n=3, 5%)	DNS	median: 22.6-26	N=64, 100%	California	urban and rural
Evans, W.D., 2012	female (N=123, 100%)	DNS	27.6	n=98, 79.7%	Virginia	urban
Frates, J., 2006	female (n=250, 50%);<br>male (n=250, 50%)	DNS	<35: n=246, 49%<br>35+: n= 253, 51%	N=500, 100%	California	urban

Freda, M.C., 1990	Female (N=615, 100%)	DNS	<18: n=21, 3%<br>18-25: n=262, 43%<br>25-30: n=115, 19%<br>31-35: n=110, 18%<br>>35: n=13, 2%<br>DNS: n=94, 15%	n=347, 56.4%	New York	urban
Gerber, B., 2005	female (n=36, 64.3%); male (n=20, 35.7%)	DNS	56.6	n=32, 57.1%	DNS	DNS
Gilliam, M., 2003	female (N=13, 100%)	Mexico (45%); US (27%); Puerto Rico (27%)	18-20: n=4, 30.8%<br>21-23: n=3, 23.1%<br>24-25: n=4, 30.8%<br>DNS: n=2, 15.4%	N=13, 100%	Illinois	urban
Goel, M.S., 2011	female (n=91, 100%)	DNS	51	N=91, 100%	DNS	DNS
Heisler, M., 2014	female (n=133, 70.7%); male (n=55, 29.3%)	Mexico (31%); Ecuador (31%); Dominican Republic (10%); El Salvador (7%); Other, non US born (21%)	51.5	n=107, 56.9%	Michigan	urban
Jerant, A., 2014	female (n=761, 65.4%); male (n=403, 34.6%)	Mexico (40.6%); El Salvador (19.4%); Honduras (21.3%); Ecuador (13.8%); Other Hispanic (3.4%)	57	n=589, 50.6%	California; New York; Colorado; Texas	urban
King, A., 2013	female (n=29, 72.5%); male (n=11, 27.5%)	"Average" country of origin: Mexico, El Salvador.	68.3	n=37, 92.5%	California	urban

Lalonde, B., 1997	Street Interview: female (n=311, 48,0%); male (n=337, 52.0%); High School Telenovela Interviews: female (n=323, 50.0%); male (n=323, 50.0%)	Mexico (51%)	Street Interview Radionovela: DNS High School Telenovela Interviews: 15	Street Interview: n=648, 100% High School Telenovela Interviews : n=395, 61.1%	Washington	rural
Leeman-Castillo, B., 2010	female (n=158, 53%); male (n=141, 47%)	DNS	31-50: n=191, 64%	N=299, 100%	Colorado	urban
Makoul, G., 2009	female (n=137, 50.7%) male (n=133, 49.3%)	Colombia (25%); Cuba (5); Dominican Republic (25%); El Salvador (10%); Guatemala (5%); Honduras (5%); Mexico (10%); Puerto Rico (15%)	59	N=270, 100%	Illinois	urban
Matthews, P.2009	female (n=16, 66.7%); male (n=8, 33.3%)	DNS	18-30: n=1, 4.2% 31-40: n=3, 12.5% 41-50: n=3, 12.5% 51-60: n=8, 33.3% 61-70: n=6, 25.0% 70+: n=3, 12.5%	n=20, 83.3%	Georgia	urban
McDonald, D., 2012	female (n=17, 94.4%) male (n=1, 5.6%)	Mexico (39.6%); Puerto Rico (31/1%); Guatemala (6.3%); USA (5.9%); Cuba (4.1%); Ecuador (3.0%); Honduras (2.6%); Colombia (2.2%); Other, South America (2.6%); Other, Caribbean (2.6%)	68.1	N=18, 100%	Connecticut	urban

Osilla, K., 2012	NA	DNS	18+	majority Hispanic	California	urban
Porter, S., 2009	DNS	DNS	DNS	N=9, 100%	DNS	DNS
Quinn, G., 2009	female (N=74, 100%)	English speaking Latino (N=10): Mexico (20%), US (80%)<br>Spanish speaking: (N=8): US (25%), Mexico (50%), Unreported (25%)	26	N=74, 100%	Florida	urban
Reuland, D., 2012	female (n=51, 63.8%); male (n=29, 36.2%)	DNS	56	N=80, 100%	North Carolina	urban
Rosas, L., 2014	female (N=152, 100%)	Puerto Rico (54%); Cuba (46%)	DNS	N=152, 100%	California	rural
Scheinmann, R., 2010	female (N=272, 100%)	Mexico (45%); Central America (21%); South America (29%); Caribbean (5%)	18–24: n=69, 25.4%<br>25–29: n=96, 35.3%<br>30–34: n=62, 22.8%<br>35+: n=45, 16.5%	N=272, 100%	New York	urban
Stockwell, M.S., 2015	female (n=327, 49.5%); male (n=333, 50.5%)	DNS	DNS	n=586, 88.8%	New York	urban
Suarez, L., 1993	female (N=376, 100%)	Mexican-American	55.8	n=209, 56%	Texas	urban
Thompson, D.A., 2012	female (n=148, 92.5%); male (n=12, 7.5%)	Mexico (76%)	27.6	N=160, 100%	Maryland	urban

Valdez, A., 2002	female (N=1197, 100%)	Mexico (73%); Peru (9%); Colombia (9%); Guatemala (9%); Ecuador (9%)	40+	N=1197, 100%	California	urban
Vaughn. S., 2012	females (N=12, 100%)	DNS	25-38	N=12, 100%	California	urban
West, A., 2014	female (n=13, 65.0%)<br>male (n=7, 35.0%)	US (70%); Argentina (8.6%); Dominican Republic (8/7%); Mexico (8.0%); Puerto Rico (4.9%)	53.9	N=20, 100%	Massachusetts	urban
Wilkin, H.A., 2007	female (n=1555, 61.8%);<br>male (n=961, 31.2%)	DNS	DNS	N=2516, 100%	Nationwide (USA)	NA
Zyskind, A., 2009	female (n=76, 70.4%)<br>male (n= 32, 29.6%)	DNS	Below 45 : n=24, 22.2%<br>45-59: n=48, 44.4%<br>60+: n= 36, 33.3%	n=69, 63.9%	Washington, DC	urban
