# Supplementary material for: Spanish-Language Consumer Health Information Technology Interventions: A Systematic Review
Source: J Med Internet Res. 2016 Aug 10;18(8):e214. doi: 10.2196/jmir.5794 (PMC4997005; doi:10.2196/jmir.5794)
Supplement: Multimedia Appendix 6 [file jmir_v18i8e214_app6.pdf]

Table 5. Intervention characteristics of included studies.

Author, Year	Technology Platform	Technology Functionality	Language of Application	Technology Delivery Location	Cultural Tailoring Category
Alcalay, R., 1999	television, radio (telenovela public service announcements)	Inform; Communicate (call in for additional info)	English; Spanish	Ubiquitous	Content (Focus groups were conducted with Latinos to discuss perception of CVD and content of campaign); User Interphase (Traditional Latino family used as actors in telenovela)
Alvaro, E., 2006	television, radio (media campaign)	Inform	Spanish	Ubiquitous	Content (Catholic Bishop of County expressed religious views within video)
Alvaro, E., 2010	television, radio (media campaign)	Inform; Communicate (encouraged calls to telephonic hotline).	Spanish	Ubiquitous	Content (Participant focus groups guided design; Incorporation of cultural factors into content)
Aragones, A., 2010	DVD	Inform	Spanish	Clinic	DNS
Arora, S., 2014	cellphone - text message	Remind/Alert; Inform	English; Spanish	Ubiquitous	DNS

Arora, S., 2015	cellphone - text message	Inform; Instruct; Record	English; Spanish	Ubiquitous	DNS
Brown, S., 1992	VCR (videotape)	Inform	English; Spanish	Clinic	Content (Inclusion of traditional values including family ties and spiritual values);<br>User Interface (Use of community religious leader and local scenes in video.)
Bolin, J., 2013	kiosk	Inform; Communicate	English; Spanish	Clinic; Community Center	DNS
Byrd, T., 2013	unspecified	Inform	English; Spanish	Ubiquitous	Content (Video uses role modeling by women from the community)
Calderón, J., 2010	unspecified	Inform; Communicate	English; Spanish	Ubiquitous	Content (Language adaptation, acknowledgement of traditional healing practices, and inclusion of family considered in video design)
Calderón, J., 2014	unspecified	Inform	English; Spanish	DNS	User Interphase (Focus group discussions led to alteration of avatar appearance and music)
Calles-Escandón, J., 2009	radio	Guide; Instruct; Record; Display	Spanish	Ubiquitous	DNS

Collins, T., 2014	cellphone - text message	Inform; Communicate	English; Spanish	Ubiquitous	DNS
de Nuncio, M., 1999	radio	Inform; Guide	Spanish	Ubiquitous	Content (Hispanic advertising firms consulted in finalization of immunization materials and mass media strategies)
Evans, W.D., 2012	cellphone - text message	Inform; Instruct	English; Spanish	Ubiquitous	DNS
Frates, J., 2006	television and radio	Inform; Communicate	English; Spanish	Ubiquitous (media campaign)	Content (Interviews conducted with community members to guide video word choice);<br>Functionality (chose radio stations with highest density of Hispanic viewers)
Freda, M.C., 1990	VCR (videotape)	Inform	English; Spanish	Clinic	DNS

Gerber, B., 2005	kiosk	Inform; Record	English; Spanish	Clinic	Content (Patient testimonials used in videos);<br>User Interphase (Video actors modeled after minority groups)
Gilliam, M., 2003	VCR (videotape)	Inform	English; Spanish	Clinic	Content (Focus groups guided design of video content; allusions to cultural messages throughout the film such as murals of the Virgin de Guadalupe and parents with children; Young Latinas speaking of their personal experience with contraceptives used as actresses)
Goel, M.S., 2011	unspecified	Inform	English; Spanish	Clinic	DNS
Heisler, M., 2014	tablet	Inform	English; Spanish	Ubiquitous	DNS
Jerant, A., 2014	computer	Inform; Guide	English; Spanish	Clinic	DNS
King, A., 2013	computer (embodied conversational agent)	Inform	English; Spanish	Community Center	Content (References to local Latino culture)

Lalonde, B., 1997	radio, television	Inform	English; Spanish	Ubiquitous and School	Content (Themes and cultural values incorporated into content of novelas based on community discussions; storyline built on a event - an alcohol related death of a high school wrestler in the community);<br>Technology Platform (Radionovela and telenovela for adults and youth, respectively, were chosen based on community discussion of preferred format);<br>User Interphase (Iterative feedback on voice was obtained during the design process)
Leeman-Castillo, B., 2010	kiosk	Inform; Reminder/Alert	English; Spanish	Clinic; Community Center (coffee shop, church, school, social service agencies)	DNS
Makoul, G., 2009	kiosk	Inform	English; Spanish	Clinic	Content (Focus groups conducted with community to tailor content of applications)
Matthews, P.2009	kiosk	Guide; Display	English; Spanish	Community Center	DNS
McDonald, D., 2012	computer (virtual pain coach video)	Inform	English; Spanish	Clinic	User Interphase (Native Spanish speaking practitioner served as actor);<br>Content: (Focus group conducted with native Spanish speakers, and minor revision made to content based on focus group feedback)

Osilla, K., 2012	computer	Inform; Record	English; Spanish	Clinic (office setting)	Content (Cultural adaptations to application included translation of idiomatic expressions e.g. getting high or feeling drunk and intervention key terms; Application provided examples of how drinking can affect the family and utilized the neutral name "Danny; low literacy methods used to convey messages)
Porter, S., 2009	computer	Inform; Instruct	Spanish	Ubiquitous	Content (Commonly occurring food items were recorded and incorporated into the Spanish version of the Web site; Diction tailored based on population feedback)
Quinn, G., 2009	unspecified (videonovela)	Inform; Guide	English; Spanish	DNS	Content (Focus groups were conducted with Mexican and Mexican-American women, to assess their preferences for existing folic acid materials and to gain an understanding of their knowledge, attitudes, and behaviors regarding birth defects and folic acid)
Reuland, D., 2012	computer	Inform	English; Spanish	Clinic	Content (Focus group conducted; themes identified were incorporated into application; Emerging themes included embarrassment about screening procedures, physicians as authority figures, familism, personalism, machismo, and language barriers)

Rosas, L., 2014	kiosk	Inform	English; Spanish	Clinic	Functionality (Touchscreen and text-to-speech software to ensure accessibility to low-literacy users; a game based on the popular Mexican Bingo game, known as Loteria, was included)
Scheinmann, R., 2010	DVD	Inform	English; Spanish	Ubiquitous	DNS
Stockwell, M.S., 2015	cellphone - text message	Remind/Alert	English; Spanish	Ubiquitous	DNS
Suarez, L., 1993	television, radio	Inform	English; Spanish	Ubiquitous	Content (Use of role models talked and looked like the target group)

Thompson, D.A., 2012	computer	Inform	Spanish	Clinic	Content (Incorporation of common beliefs and practices within the target population; text, images and audio that account for the educational levels and health literacy); User Interphase (audio used voice of a native Spanish-speaking woman of Mexican origin)
Valdez, A., 2002	kiosk	Remind	English; Spanish	Clinic	Content (Explanation about disease presented in the context of "real life" concerns of Latinas)
Vaughn. S., 2012	television (telenovela)	Inform; Display	Spanish	Clinic	Content (Video included local scenes and actors)
West, A., 2014	tablet	Inform	Spanish	Clinic	Content (Use of actors that looked to be of Latino origin; filming within local hospital setting)
Wilkin, H.A., 2007	television (telenovela)	Inform	Spanish	Ubiquitous	DNS

Zyskind, A., 2009	computer (Internet video through MedlinePlus)	Inform	English; Spanish	Clinic	DNS
