# Supplementary material for: Spanish-Language Consumer Health Information Technology Interventions: A Systematic Review
Source: J Med Internet Res. 2016 Aug 10;18(8):e214. doi: 10.2196/jmir.5794 (PMC4997005; doi:10.2196/jmir.5794)
Supplement: Multimedia Appendix 7 [file jmir_v18i8e214_app7.pdf]

Table 6. Outcome metrics, study design, and evaluation results of included studies.

Author, Year	Outcome Metrics (Specific)	Study design	Evaluation Results
Alcalay, R., 1999	Knowledge Retention (cardiovascular disease prevention and risk factors)	Non-experiment; Pretest-Posttest	Knowledge increase. No change in current health behaviors.
Alvaro, E., 2006	Behavior/Attitude Change (beliefs/attitudes about organ donation)	Quasi-experiment; Non-equivalent groups; Pretest-Posttest	Substantial exposure of target audience to media intervention. Increase in pro-donation beliefs and family discussion on donation post intervention.
Alvaro, E., 2010	Behavior/Attitude Change (awareness of organ donation and Behavior intentions to donate)	Quasi-experiment; Non-equivalent groups; Pretest-Posttest	Increase in intention to be a living organ donor within intervention group.
Aragones, A., 2010	Behavior/Attitude Change (colorectal cancer screening rates)	Randomized Experiment; Posttest	Significant difference in overall rate of completed screening for colorectal cancer in intervention group versus control group.
Arora, S., 2014	Behavior/Attitude Change (use of emergency department, healthy behaviors; medication adherence; self-efficacy; performance of self-care tasks); Biometric Marker (HbA1C levels); Knowledge Retention (on diabetes); Usability (opinions on the types and frequency of text messages, message content, and willingness to recommend program to family)	Randomized Experiment; Pretest-Posttest	HbA1C level decreased by 1.05% in the intervention group compared with 0.60% in the controls (Δ0.45; 95% confidence interval [CI] – 0.27 to 1.17) at 6 months.
Arora, S., 2015	Behavior/Attitude Change (appointment adherence)	Randomized Experiment; Posttest	Significant difference in appointment adherence rate between experimental and control groups.

Bolin, J., 2013	Behavior/Attitude Change (intent to change behavior);<br>Usability (reaction to the kiosk)	Non-experiment;<br>Posttest	Diosks received 5,300 uses in 11 months of study. Nearly 90% of diosk users believed that the diosk would help them take better care of their diabetes, whereas 85% planned to make changes in their lifestyle.
Brown, S., 1992	Knowledge Retention (diabetes knowledge test); Usability (videotape acceptability)	Quasi-experiment;<br>Non-equivalent groups;<br>Pretest-Posttest	Moderate effect size in difference in knowledge scores between control and experimental group.
Byrd, T., 2013	Behavior/Attitude Change (Papanicolaou test screening)	Randomized Experiment; Pretest-Posttest	Women in any of the intervention arms were significantly more likely to report being screened than those in the usual care group in two of three study sites.
Calderón, J., 2010	Behavior/Attitude Change (self-reported breast self-exam and mammography screening rates);<br>Knowledge Retention (breast cancer knowledge)	Quasi-experiment;<br>Non-equivalent groups;<br>Pretest-Posttest	No significant difference was observed between groups at baseline or post-intervention.
Calderón, J., 2014	Usability (cultural and linguistic appropriateness);<br>Knowledge Retention (diabetes health literacy)	Randomized Experiment; Posttest	Significant difference in improvement of diabetes health literacy scores between experimental and control groups.
Calles-Escandón, J., 2009	Behavior/Attitude Change (listenership / use of the media as a source of health information);<br>Knowledge Retention (general medical knowledge)	Non-experiment;<br>Pretest-Posttest	Pronounced increase in radio listenership and use of media as a source of health information post-intervention.
Collins, T., 2014	Behavior/Attitude Change (exercise behaviors)	Non-experiment;<br>Pretest-Posttest	Nearly all participants progressed from the contemplation stage to the action/maintenance/termination stage of exercise readiness.

de Nuncio, M., 1999	Knowledge Retention (knowledge of basic immunization schedule);<br>Usability (overall approval of both commercials, message clarity, educational value and emotional appeal, and speed)	Non-experiment;<br>Pretest-Posttest	Commercials rated favorably on qualitative questions regarding message clarity, speed, amount of information, and educational value. Limited change in knowledge after intervention.
Evans, W.D., 2012	Behavior/Attitude Change (health promoting and risk avoidance behaviors including alcohol consumption during pregnancy, pre-natal care visits, diet, and vitamins consumption);<br>Self-reported Health (preparedness for motherhood);<br>Usability (cognitive and affective reactions to text4baby messages)	Randomized Experiment;<br>Pretest-Posttest	Significant increase in self-expressed preparedness for motherhood and improvement of attitudes toward alcohol consumption from baseline to follow-up.
Frates, J., 2006	Behavior/Attitude Change (organ donation attitudes and behaviors)	Non-experiment;<br>Pretest-Posttest	Measures of organ donation attitudes and behaviors (decision and declared intent to donate organs) improved significantly in 2001 and 2002, then leveled off or declined in 2003.
Freda, M.C., 1990	Knowledge Retention (test concerning preterm birth, specifics of instrument not provided)	Non-experiment;<br>Pretest-Posttest	Significant knowledge transfer and retention through postpartum period for patients viewing the videotape.
Gerber, B., 2005	Behavior/Attitude Change (compliance with eye examinations);<br>Usability (ease of use)	Quasi-experiment;<br>Non-equivalent groups;<br>Pretest-Posttest	No change in attitude toward diabetic eye disease detected. Most individuals felt the multimedia was 'easy' or 'very easy' to use.
Gilliam, M., 2003	Usability (video length, format, and identification with video actors)	Non-experiment;<br>Posttest	Participants responded positively to video content and to the use of culturally relevant messages.
Goel, M.S., 2011	Knowledge Retention (knowledge of breast cancer prevention techniques);<br>Behavior/Attitude Change (attitude towards breast cancer and screening)	Non-experiment;<br>Pretest-Posttest	Significant knowledge increase from pretest to posttest. There were no changes in mean attitudes, which were high at baseline.

Heisler, M., 2014	Behavior/Attitude Change (medication adherence);<br>Biometric Marker (HA1C level);<br>Knowledge Retention (antihyperglycemic medications);<br>Self-reported Health (diabetes distress, self-efficacy);<br>Usability (satisfaction with antihyperglycemic medication information)	Randomized Experiment; Pretest-Posttest	iDecide participants reported significantly greater improvements in satisfaction with medication information and in diabetes distress compared to the print material group. There were no differences between groups in other outcomes.
Jerant, A., 2014	Behavior/Attitude Change (screening completion, self-efficacy, and colonoscopy stage of readiness);<br>Knowledge Retention (knowledge of screening test options and screening risks)	Randomized Experiment; Pretest-Posttest	Significant increase in colorectal cancer screening knowledge, self-efficacy, readiness, test preference specificity, discussion, and recommendation compared to control group.
King, A., 2013	Behavior/Attitude Change (increase in walking);<br>Usability (credibility and acceptability of program)	Randomized Experiment; Pretest-Posttest	Significant difference in walking behavior in the intervention group versus control group.
Lalonde, B., 1997	Behavior/Attitude Change (attitude toward alcohol);<br>Usability (perception of telenovela)	Non-experiment; Pretest-Posttest	Significant but small improvement in summary alcohol attitude scores among youth in post viewing group. Focus groups thought radio drama was interesting and presented good messages. Less than 10% of random street interviewees had seen telenovela. Twice this percentage has heard radionovela.
Leeman-Castillo, B., 2010	Behavior/Attitude Change (nutrition and physical activity, measured by meeting recommended guidelines, and smoking behavior)	Non-experiment; Pretest-Posttest	Authors found significant improvements in nutrition and physical activity among Latino program users; however, no changes were observed with respect to smoking behaviors.

Makoul, G., 2009	Behavior/Attitude Change (willingness to consider screening options and intention to discuss CRC screening with the doctor);<br>Knowledge Retention (anatomy, key terms, and knowledge of primary screening options);<br>Usability (reactions to the multimedia patient education program, e.g. believable, reassuring, etc.)	Non-experiment; Pretest-Posttest	Significant increase in knowledge of anatomy and key terms, primary screening options, and risk information as well as willingness to consider screening.
Matthews, P., 2009	Behavior/Attitude change (intent to follow up with healthcare professional; lifestyle and diet changes);<br>Usability (perceived utility and knowledge gain; characteristics influencing kiosk)	Non-experiment; Posttest	Participants reported kiosk to be a useful source of information. Online survey respondents indicated intention to change diet or exercise habits.
McDonald, D., 2012	Self-reported Health (pain intensity, depressive symptoms, and treatment changes)	Randomized Experiment; Pretest-Posttest	No significant group difference for pain intensity, pain interference with activities, or depressive symptoms 1 month later.
Osilla, K., 2012	Usability (acceptability and feasibility of in-person feedback sheet, appeal of messages, and cultural acceptability)	Non-experiment; Posttest	Application was reported to be engaging, interactive and personal. Spanish-speaking clients felt less shame, embarrassment, and discomfort with the web-MI compared to other in-person groups.
Porter, S., 2009	Knowledge Retention; (diabetes-related knowledge, carbohydrate-counting skills, ability to plan a meal via the plate method);<br>Usability (usefulness, cultural appropriateness, readability, and ease of access)	Non-experiment; Pretest-Posttest	Knowledge increase for almost all questions post intervention.
Quinn, G., 2009	Behavior/Attitude Change (intention to engage in preventative behaviors);<br>Knowledge Retention (folic acid use and benefits);<br>Usability (reaction to education material and cultural appropriateness, readability)	Quasi-experiment; Non-equivalent groups; Pretest-Posttest	After exposure to the campaign materials, all of the women seemed to understand the purpose of the materials, were able to clearly state the message and “call to action” of the campaign and 93% said they would begin to take vitamins.

Reuland, D., 2012	Knowledge Retention (screening knowledge);<br>Behavior/Attitude Change (intent to obtain and completion of screening)	Non-experiment; Pretest-Posttest	Knowledge scores and self-efficacy were increased immediately after program viewing. Of the 85% of participants completing the four-month follow up survey, 53% of participants reported discussing screening with a provider and 19% completed a test.
Rosas, L., 2014	Knowledge Retention (pre/post test on environmental health);<br>Usability (opinions on information, voice, language graphics, and ease of use)	Non-experiment; Pretest-Posttest	Significant knowledge gain at posttest. Over 90% of women reported that they learned something new while using the kiosk.
Scheinmann, R., 2010	Behavior/Attitude Change (self reported infant feeding behavior);<br>Knowledge Retention (knowledge of infant feeding)	Quasi-experiment; Non-equivalent groups; Pretest-Posttest	Knowledge increased for both groups, but the video group showed a greater increase in knowledge between baseline and 6 months. The video group also showed positive changes in behavior, including older age of child at first solid feeding.
Stockwell, M.S., 2015	Behavior/Attitude change (receipt and timeliness of second dose of influenza vaccine in children);<br>Usability (telephone satisfaction survey)	Randomized Experiment; Posttest	Children in the educational arm were more likely to receive a second vaccine dose by April 30 (72.7%) versus conventional (66.7%) versus written reminder-only arm (57.1%; P = .003). They also had more timely receipt by day 42 ($P < .001$) and over time ($P < .001$).
Suarez, L., 1993	Behavioral/Attitude Change (breast and cervical cancer screening rate and attitude)	Non-experiment; Pretest-Posttest	Suggested increase in mammogram use. Pap smear use was not substantially improved.
Thompson, D.A., 2012	Behavior/Attitude change (changes in infant/toddler feeding patterns and interaction with physicians);<br>Knowledge Retention (infant/toddler nutrition and feeding)	Randomized Experiment; Posttest	Significant difference in mean total summed knowledge score between intervention and control groups.
Valdez, A., 2002	Behavior/Attitude Change (attitude toward breast cancer and mammography intentions);<br>Knowledge Retention (breast cancer knowledge)	Randomized Experiment; Posttest	Women in the intervention group exhibited significantly higher knowledge scores than the pretest group. The intervention also increased the likelihood of women seeking information about a mammogram.

Vaughn. S., 2012	Behavior/Attitude Change (purchase and preparation of food);<br>Usability (how to improve telenovela)	Non-experiment; Posttest	Participants demonstrated basic knowledge between food and health. Positive qualitative feedback was gathered on usefulness of video.
West, A., 2014	Knowledge Retention (general anesthesia);<br>Self-reported Health (self reported anxiety and satisfaction with preoperative anesthesia process)	Randomized Experiment; Posttest	Significant reduction in anxiety score and increase in satisfaction in patients who viewed the video compared with those who did not. No difference in knowledge improvement between the two groups.
Wilkin, H.A., 2007	Behavior/Attitude change (intention to get or recommend mammograms);<br>Knowledge Retention (recommendations for treatment)	Quasi-experiment; Pretest-Posttest	Significant increase in knowledge regarding breast cancer among viewers. No significant change in behavioral intention to receive a mammogram.
Zyskind, A., 2009	Behavior/Attitude Change (use of online health resources);<br>Biometric Marker (Hemoglobin A1c levels, LDL cholesterol, random blood glucose, and total cholesterol);<br>Knowledge Retention (knowledge about diabetes)	Randomized Experiment; Pretest-Posttest	The intervention group had a small decline in HbA1c and LDL cholesterol, while the control group had a small increase in both of these measures.
